# The current techniques in dorsal augmentation rhinoplasty: a comprehensive review

**DOI:** 10.1186/s40902-024-00418-9

**Published:** 2024-04-28

**Authors:** Nariman Nikparto, Amir Yari, Saeed Hasani Mehraban, Meysam Bigdelou, Amirali Asadi, Amirmohammad Arabi Darehdor, Sayna Nezaminia, Mehdi Khani, Lotfollah Kamali Hakim, Fateme Eskandari, Maryam Erfani, Hamid Tebyaniyan

**Affiliations:** 1 Department of Oral and Maxillofacial Surgery, School of Dentistry, Zanjan University of Medical Sciences, Zanjan, Iran; 2 Department of Oral and Maxillofacial Surgery, School of Dentistry, Kashan University of Medical Sciences, Kashan, Iran; 3 Department of Oral and Maxillofacial Surgery, School of Dentistry, Tehran University of Medical Sciences, Tehran, Iran; 4 Oral and Maxillofacial Surgeon, Tehran, Iran; 5 Department of Oral and Maxillofacial Surgery, School of Dentistry, Alborz University of Medical Sciences, Karaj, Iran; 6 Resident of Oral and Maxillofacial Surgery, Department of Oral and Maxillofacial Surgery, School of Dentistry, Tehran University of Medical Science, Tehran, Iran; 7 Student Research Committee, School of Dentistry, Shiraz University of Medical Sciences, Shiraz, Iran; 8 Department of Science and Research, Islimic Azade University, Tehran, Iran

**Keywords:** Dorsal, Augmentation, Rhinoplasty, Surgical approaches

## Abstract

**Background:**

An essential aspect of rhinoplasty is the enhancement of the nasal dorsal contour by performing dorsal augmentation (DA) rhinoplasty. A wide range of techniques are available for DA as the demand for aesthetic nasal refinement grows. This review aims to provide a comprehensive overview of the current techniques used in DA rhinoplasty.

**Main body:**

Research articles on DA rhinoplasty techniques were identified through a comprehensive literature search. Scopus, PubMed, and Web of Science were used as electronic databases. Each database was searched for articles published since its inception. DA rhinoplasty techniques were examined in this literature review. Methodological quality was assessed for the selected studies, and data was extracted to examine materials used, surgical approaches, and reported outcomes for each technique. Various DA methods, including autologous grafts and synthetic implants, are examined in-depth in this review. Comparing approaches can help better understand their respective advantages and limitations.

**Conclusion:**

A wealth of techniques is available for DA rhinoplasty, each with advantages. Patients’ nasal anatomy, desired outcomes, and potential risks must be considered by surgeons when determining their surgical approach. DA methods continue to evolve rapidly, creating a need for a thorough understanding of the current landscape to make informed decisions.

## Background

Historically, sterile paraffin was used to augment saddle nose deformities in the late nineteenth century as part of rhinoplasty procedures. As soon as DA was discovered, it became evident that it requires great precision and optimal graft materials to be performed. Bird bone, pork cartilage, and cow leather were early materials used for nasal augmentation. Modern surgical techniques emerged after Konig used autogenous cartilage to reconstruct nasal deformities. DA has remained challenging in rhinoplasty despite developing various augmentation strategies and graft materials over the past century. DA techniques have become increasingly common in recent decades as reductive rhinoplasty has lost some of its luster, and preference has shifted to augmentation, especially in ethnic rhinoplasties. Reconstructive surgery was the first use of dorsal augmentation, aiming to correct inborn and acquired deformities [1–6]. Prominent facial features, such as the nasal dorsum, characterize faces. The facial aesthetic unit will blend in if it is symmetrical and well-proportioned. Visually deforming and asymmetrical facial features can be visually striking if they are not symmetrical, irregular, or disproportionate. There is a thin layer of soft tissue over the rhinion of the nose. DA is, therefore, one of the most complication-prone rhinoplasty procedures because any irregularity will be visible and may sully the outcome [1, 7]. Preoperative planning involves assessing the size and length of nasal bones, assessing for pathology, and determining the optimal location and degree of augmentation based on physical examination findings and photographic analysis. These calculations should also be used to lengthen the nose or increase tip projection. DA in rhinoplasty aims to make the nose fit the face with an appropriate dorsum and aesthetic lines [1, 3, 7, 8]. In primary and revision procedures, Rhinoplasty specialists use DA as an essential tool. There are intrinsic and acquired causes contributing to nasal dorsum deficiencies. It is common for Asians and blacks to have a low dorsal height. Also, infections, traumas, and previous rhinoplasty can lead to a deficient osseocartilaginous framework. Many revision patients present with irregular contours resulting from supranasal grafts or structural elements under the skin envelope and over-resection of the nasal dorsum. Dorsal augmentations are often recommended for these cases due to their aesthetic and structural appeal. Various sources have been used for dorsal augmentation, including autologous, allogeneic, and synthetic sources [9–15]. Autologous options include bone, cartilage, fascia, perichondrial, diced cartilage, and dermofat grafts. Several allogeneic and synthetic sources exist, including allogeneic cartilage, bone, and dermis [2]. A well-planned and communicated preoperative plan and good communication with the patient are vital for augmentation rhinoplasty. The purpose of this surgery was described using a variety of surgical methods. The modified autologous dorsal nasal augmentation rhinoplasty technique uses an autologous PRP carrier. Diced cartilage and fascia grafts are used in this technique, modified from Daniel and Calvert’s. Increasingly, aesthetic purposes have been the focus of augmentation over the last few decades [9, 10]. As a result of previous unsuccessful rhinoplasties resulting in graft failure or resorption, secondary revision rhinoplasties have also become more familiar to correct previous failed attempts, so innovators are being forced to find the best graft materials that have reduced associated morbidity and improved handling characteristics. In alloplastic and autogenous grafts, the lines are often blurred between aesthetic and reconstructive grafting procedures. Creating pleasing dorsal aesthetic lines is the goal of DA rhinoplasty. To maintain high patient satisfaction, any technique must have a low rate of complications. To achieve this, the surgeon must master multiple techniques, analyze each patient's anatomy and goals thoroughly, and have much knowledge about different graft and implant options [16–18]. According to most experts in the field, there is no such thing as an ideal graft [1, 10, 19–22]. This review discusses a comprehensive overview of the current techniques employed in DA rhinoplasty in recent decades.

## Method

A comprehensive literature search was conducted to identify relevant articles on DA rhinoplasty techniques. The following electronic databases were utilized: Scopus, PubMed, and Web of Science. Inclusion criteria encompassed articles written in English, and the search terms used were a combination of keywords and MeSH, including “graft,” “dorsal augmentation,” “rhinoplasty,” and “surgical techniques.” Studies on surgical and non-surgical approaches, including implants, grafts, and injectables, were considered. The synthesis included a thematic analysis of the various DA techniques, highlighting their advantages, limitations, and reported outcomes. Exclusion criteria encompassed articles not written in English, case reports lacking substantial discussion on techniques, and studies with inadequate methodological detail. Key themes and patterns across studies were identified to provide a comprehensive overview of the current state of DA rhinoplasty approaches.

### Who needs the DA rhinoplasty?

As a surgical procedure that aims to enhance the appearance of the nasal bridge, DA rhinoplasty is also known as dorsal hump reduction or augmentation rhinoplasty. Rhinoplasty that produces a visible bump or convexity on the nasal bridge is usually recommended for individuals with a dorsal hump. People with prominent dorsal humps often seek DA rhinoplasty to improve the profile of their nose. It could be genetic, trauma-related, or as a result of other factors. Commonly, the procedure is used to improve facial harmony and address aesthetic concerns. If candidates are considering DA rhinoplasty, they should consult with an experienced plastic surgeon or otolaryngologist. An evaluation of the nasal anatomy, a discussion of the individual's aesthetic goals, and consideration of their overall health are all required before the procedure can be performed [1, 4, 5, 15, 23, 24]. There are several reasons why augmentation of the nose may be necessary: dorsal hump or nasal bump: a dorsal hump or nasal bump on the nasal bridge is one of the primary indications for augmentation of the nose. An aging process, trauma, or genetics can cause this hump. A prominent dorsal hump can be smoothed and more aesthetically pleasing with this procedure for individuals who are unhappy with the appearance of the dorsal hump. DA rhinoplasty is commonly performed for cosmetic reasons. The overall harmony and balance of facial features can be significantly affected by a dorsal hump. Several studies have shown that this procedure can greatly enhance facial symmetry and better complement the remaining facial proportions [2, 11, 25–29]. It can be challenging to feel confident and self-esteem when people have aesthetic concerns about their noses. It is possible for patients to feel more self-confident and have a better quality of life after undergoing DA rhinoplasty due to the improved appearance of their face. An obstruction or difficulty breathing may occur as a result of a dorsal hump. As well as improving the appearance of the nasal structure, DA rhinoplasty can improve its functionality by ensuring that it is both functional and aesthetic in design. In deciding to undergo cosmetic surgery, a patient's preferences are crucial. Even though a dorsal hump does not cause functional problems for some people, they may find it bothersome. It can shape the nose to fit aesthetic preferences by having DA rhinoplasty [5, 6, 8, 23, 30–34].

### Grafts and surgical approaches

Many rhinoplasty surgeons believe that autologous grafts are the best material for grafting. Patient acceptance and low infection rates make autologous grafts a great choice. However, autologous grafts have several well-known disadvantages, including irregular shapes, absorption, donor site morbidity, and aesthetic results that may preclude use in revision or more extensive surgeries [31]. Table 1 summarizes the current technique studies of dorsal augmentation rhinoplasty. Following are explanations of various grafts and surgical techniques.

**Table 1:** The current technique studies dorsal augmentation rhinoplasty

Aim	Method	Outcomes	Ref/year
Nasal dorsum grafts are carefully stitched, filled, and fixed to maximize predictability.	An extension of the septal cartilage dorsum border is fixed to the septal cartilage graft to increase nasal dorsum height. Surgical outcomes and operative courses were analyzed for patients the senior author treated with this operative technique. Case examples illustrate the indications and outcomes of the procedure.	Patients with under-projected noses can benefit from this grafting technique by improving functionality, defining dorsal aesthetic lines, and achieving a more balanced profile.	[4]/2023
A rabbit model was used to investigate using a HA matrix as an allograft.	Eight rabbits were osteotomized. Four animals underwent a sham operation as a control group, while four received a saline-gelled HA matrix and cartilage slices.	DA rhinoplasty may be improved with the HA matrix. Membranous ossification was accompanied by collagen development; however, complete ossification requires more time.	[35]/2023
Using this improved method, the authors assessed the safety and efficacy of augmentation rhinoplasty in these patients.	During the study, 52 thin-skinned Asian noses with modified perichondrium on dorsal onlay grafts underwent open augmentation rhinoplasty. Evaluation of aesthetic outcomes was performed using the ROE scale.	In long-term follow-up, ROE scores were statistically different before and after surgery. Approximately 97% of patient satisfaction was reported during and after surgery on the ROE scale, while 20.65% were reported as satisfied postoperatively. A high satisfaction rate was reported by patients for the nasal dorsal improvement using the VAS questionnaire. Fat liquefaction and warping occurred at the donor site, but no other serious complications occurred. The best method to conceal the nasal dorsum of thin-skinned patients is to perform an onlay DA rhinoplasty with perichondrium covering both edges of the graft.	[36]/2023
This study examines and illustrates his experience and outcomes in performing primary and revision rhinoplasty with PAFG.	Several prospective bicentric studies were conducted on patients with slight dorsal deficiencies undergoing rhinoplasty following hump resections, trauma, or previous rhinoplasty. After surgery, MRIs were used to measure graft resorption objectively. ROE scores were compared preoperatively and 1-year postoperatively to investigate patient satisfaction. Pair-wise t-tests were used to compare scores following normal distributions.	Rhinoplasty done with PAFG is reliable for minor augmentation and camouflaging of the dorsal aspect. Only a small amount of learning is required for the procedure.	[7]/2023
This study investigated how buried thread nasal augmentation affects the dorsal soft tissue of the nose and the result of revision rhinoplasties.	Patients requested revision rhinoplasty after buried thread nasal augmentation. A polytetrafluoroethylene expansion was used for revision rhinoplasty in the rest of the patients; autologous rib cartilage and alar cartilage were used to reshape the tip of the nose in 16 cases, and autologous septal cartilage and alar cartilage in 10 other cases.	The subcutaneous tissue of the nasal dorsum is impacted by soft tissue compliance after implantation of the absorbable thread. Absorption and degradation of the thread stimulate inflammatory cells and fibroblasts to move into the surrounding tissue and cause scarring, which can affect revision rhinoplasty design and outcome.	[37]/2023
Secondary rhinoplasty with cross-linked ADM was investigated for surgical outcomes and complications.	Between 2015 and 2018, the authors prospectively examined 56 patients who underwent secondary rhinoplasty in their clinic. Silicone implants, capsules, scar tissue removal, ADM dorsal augmentation, and autogenous cartilage tip plasty were performed in all cases. Preoperatively, 6 months postoperatively, and over one year postoperatively, the Modified Rhinoplasty Outcome Evaluation was used to assess outcomes.	After primary rhinoplasty, ADM cross-linked human DA and autogenous cartilage nasal tip work can resolve various problems caused by silicone implants. The surgical outcome was favorable, with a low infection rate, firm implant attachment, good skin texture/thickness, and gain of desired height and dorsal line.	[38]/2023
Clinical and 3D morphometric analyses were conducted to determine if septal extension grafts improved nasal and tip deformity and achieved a standard profile.	An analysis of 194 consecutive cases of unilateral cleft was performed. The septal extension graft was used in all secondary open rhinoplasty procedures between 2013 and 2021. A 3D morphometric measurement and clinical data were collected.	In this study, researchers found a significant improvement in deformities of the under-projected, up-rotated, deviated, and poorly defined nasal tips and nose bridges. Patients with cleft lip nasal deformities could benefit from the technique by having a nose more like a normative nose.	[39]/2023
UDCWF graft resorption rate and thin rib cartilage graft stability were measured.	The number of patients who underwent septal extension grafting and nasal dorsum augmentation between 2017 and 2020 was 53. Before, immediately after, and after surgery, three-dimensional photogrammetry was used to measure nasal tip height, sellion height, and nasolabial angle.	Instability and rotation were both maintained by thin rib cartilage. For Asians with thick skin and short noses, it is an effective alternative to rhinoplasty. The UDCWF graft also showed a resorption rate of approximately 7.5%, meaning a lower probability of stepping and dorsal irregularities.	[15]/2023
Primary and secondary cases aimed to achieve ideal dorsal aesthetic lines rather than adding volume with fascia and DC-F.	Four configurations of DC-F grafts were used in this study: double layer, single layer, partially filled DC-F, and full-length DC-F grafts. To prevent graft displacement, dimensions were carefully determined and sutured to the dorsum at 10 points.	It was possible to conceal irregularities in the nasal dorsum, highlight aesthetic lines, and enhance the look of nasal parts using autogenous deep temporal fascia, rectus abdominis fascia, and DC-F. Nasal dorsum grafts are carefully stitched, filled, and fixed to maximize predictability.	[40]/2022
Rhinoplasty can be camouflaged and augmented using cartilage chips, reveals this study.	The study analyzed 64 cases of rhinoplasty that were performed from 2014 to 2019. A total of 49 rhinoplasties were performed, with 15 revisions. Cartilage chips were cut into smaller pieces from 2 to 10 mm thick. They were used to fill deep radix depressions around the grafts and prevent them from being visible at the tip. The fascia was also augmented with them. Forty-six cartilage chips, 16 rib chips, and 1 ear chip were sculpted from septal cartilage.	Using cartilage chips as a camouflage and augmentation method is proving very effective.	[41]/2022
This study used ADM to compare the effectiveness of DA rhinoplasties performed as a primary procedure versus a revision procedure.	A retrospective cohort study design was used to recruit DAR patients operated on by a single surgeon over 65 months. Postoperative changes in dorsal and radix heights compared to nasal length were the primary outcomes, as well as patients’ and surgeons’ satisfaction with results on both aesthetics and function. Demographic, surgical, and pathological variables were grouped into three categories.	Although patients in this study differed significantly in age, the number of osteotomies and tipplasties, and hump reduction surgeries, ADM was found to be a safe and effective option for primary and revision DAR.	[42]/2022
Alloplastic dorsum augmentation surgery is the subject of these meta-analyses.	Duplications were removed, and 491 titles and abstracts remained. Observational studies, retrospective studies, and case series were included. The systematic review and meta-analysis included 3803 cases.	PTFE, HDE, and silicone were the most widely used alloplasts. Using a random effects model, the revision rate was 6.40% with 95%CI.	[23]/2022
Analyzing long-term complications after diced cartilage grafting for DA rhinoplasty	Infection, overcorrection, visible irregularities, absorption, and revision rates were pooled in a meta-analysis.	DA rhinoplasty complications are presented for the first time in this meta-analysis. A common complication was infection. A diced cartilage packing method did not correlate with irregularity and revision surgery.	[43]/2022
This study examined an autologous material that doesn't disperse for use in dorsal nasal augmentation. Resorption, warping, and wrapping membrane resistance are significant for this material.	An ear concha cartilage and perichondrium with fascia were removed from 30 patients. A posterior soft tissue attachment was used to say the concha cartilage. DA material was being developed.	This technique uses diced cartilage attached to the perichondrium for onlay nasal augmentation. A shell cartilage with a peculiar shape can make a flexible, versatile, and durable material.	[44]/2022
An investigation will be made into the indications, sites, techniques, complications, and patient satisfaction with temporalis fascia grafts in rhinoplasty.	From 2015 to 2020, the King Abdulaziz University Hospital in Saudi Arabia conducted this retrospective cohort study. Different forms of temporalis fascia were predictor variables. In addition to satisfaction, irregularities in the dorsal nasal region and contour definitions were reported. The reason for surgery, the surgical type, graft size, and surgery site were also considered. DA findings have been evaluated by a doctor other than a surgeon.	As a preferred nasal reconstruction material for rhinoplasty, the temporalis fascia is superficial to harvest and can be shaped in various ways.	[45]/2022
Researchers present here a new comma-shaped columellar strut graft design. Grafts serve multiple functions, including supporting the tip, modifying angles, and influencing the columella-tip relationship.	In total, 78 patients had primary cases; the rest had secondary cases. Comma strut and spreaders were used to reconstruct the cartilaginous framework.	Providing reliable support for the nasal tip, the comma strut defines the lobular-columellar angle and modifies the supratip break.	[46]/2021
Researchers present a unique technique for reconstructing Mohs defects using diced cartilage grafts and folded paramedian forehead flaps.	Recurrent basal cell carcinoma of the nose presented in a 54-year-old female who had been previously resected three times. A through-and-through nasal defect was achieved with Mohs surgery. An underlying paramedian flap was folded and staged. To enhance the dorsal area, diced cartilage, and fibrin glue were used with temporalis fascia to reconstruct the supratip break.	A paramedian forehead flap was used for nasal reconstruction using a diced cartilage graft. Rebuilding and defining the nasal dorsum can be accomplished with this technique.	[47]/2021
Bilateral septal extension struts are a new graft design. Two cartilage grafts are bilaterally fixed to the septum in a fan shape. An extension graft and a columellar strut are combined to form a “sandwich” structure.	Augmentation rhinoplasty using bilateral septal extension struts was performed on 52 female patients, ages 18 to 37.	Overall, patient satisfaction with the nose improvement was high based on their evaluations. Graft extrusion or infection was not reported. Long-term follow-up revealed severe asymmetry in two revision cases due to columellar deviation and warped dorsal onlay grafts. Strut graft warping can be mitigated with bilateral septal extension struts, making it easier to manage. Long-term support is provided to the lateral cartilages at the tip and lower end.	[48]/2021
Researchers compared outcomes for septorhinoplasty patients receiving autologous cartilage, IHCC, and Tutoplast grafts.	Consensus resolved conflicts after a dual review of abstracts and full texts. Ensure homogeneity of the study sample by including only patients undergoing en bloc dorsal onlay grafts. Five hundred seventy-six unique citations remain after removing duplicates.	In rhinoplasty patients undergoing dorsal augmentation, no differences in outcomes were observed in warping, infection, contour irregularity, resorption, or revisions between homologous and autologous costal cartilage grafts. In augmentation rhinoplasty, dorsal onlay grafts provide the nasal dorsum with structure and contour.	[49]/2020
Using objective and subjective assessment criteria, researchers compared aesthetic outcomes and complications of MCG with those of OCGs.	The DA of 82 consecutive patients was performed by a single surgeon using OCG or MCG. An anthropometric analysis and a consensus decision were used to determine the aesthetic outcomes. Additionally, postoperative complications and patient satisfaction were examined.	When MCG is used for dorsal augmentation, the aesthetic results are similar, but there is a lower rate of warping than when OCG is used. For Asian rhinoplasty, MCG might be an effective alternative to graft warping in nasal dorsal augmentation.	[27]/2020
A septal extension graft combined with mild rasping could be an alternative approach.	From March 2012 to July 2015, patients who had hump nose correction with rhinoplasty were recruited for this retrospective study. In limited cases, researchers used smooth dorsal contouring instead of conventional DA after humpectomy. A three-dimensional photogrammetric analysis was performed on 15 patients.	It is, therefore, more meaningful to balance the nasal tip and nasal dorsum after hump resection in Asians than to augment the dorsum.	[14]/2020
Comparing dice cartilage wrapping with temporalis fascia and alloderm for DA of the nose was the purpose of this study.	They randomly assigned 50 patients who needed nasal augmentation to two equal groups in a clinical trial. Diced cartilage was wrapped using temporalis fascia in the first group and a thin sheet of alloderm in the second. Two groups of patients and experts were surveyed after one year. Two groups were also compared on mean dorsal height increase.	In augmentation of the nasal dorsum, temporal fascia was more effective than alloderm in covering diced cartilage. A temporal fascia group had higher patient satisfaction and mean dorsal height.	[50]/2020
In a novel method of dorsal augmentation, the researcher combined autologous costal grafts with septal extension grafts.	This study retrospectively reviewed 28 records of augmentation rhinoplasty performed using their novel technique. In addition, researchers have shown the use of septal extension grafts to augment bony dorsums and cartilaginous dorsums. To evaluate surgical outcomes, 15 facial photographs were analyzed for anthropometric parameters.	All nasal parameters were successfully augmented. All patients had a mobile and comfortable nasal tip. Twelve patients were delighted, ten were satisfied, and six needed revision surgery. Due to visible or warped solid septal extension grafts, ten patients had caudal deviations. In Asian patients, it is possible to maximize DA by using autologous costal grafts combined with septal extension grafts in augmentation rhinoplasty.	[51]/2019
Patients with a history of rhinoplasty were evaluated for the effectiveness of closed reduction versus conservative treatment of nasal bone fractures.	Among 17 patients who underwent rhinoplasty for nasal bone fractures, five underwent a closed reduction, and 12 underwent a conservative procedure. The esthetic improvement of three of 12 conservatively treated patients was achieved with secondary rhinoplasty. Based on the modified Murray classification, all patients were classified based on fracture site and presence of a nasal septal fracture, and their disease course was analyzed.	The risk of traumatic capsular rupture is minimal, and closed reductions are recommended only when visible deviations exist.	[28]/2019
The researchers described their primary and secondary experiences with diced conchal cartilage wrapped in retroauricular fascia.	Nineteen patients with this technique had their clinical records reviewed. The entire shell was harvested and diced using the same incision, then wrapped in retroauricular fascia. The mastoid dead space was closed with quilting resorbable sutures. Researchers have used the graft in all cases using a closed approach.	Dorsal augmenting with diced cartilage has become one of the most popular procedures. With posterior auricular fascial graft, diced conchal cartilage can be wrapped and reattached to the ear without a secondary donor site, which speeds up the process and conceals the scar. Even when bilaterally harvested, costal diced cartilage wrapped in rectus abdominis fascia has several disadvantages, including a smaller amount of cartilage and a more prolonged postoperative swelling than temporal fascia.	[25]/2019
The researchers used soft tissue filler to examine the dorsal nasal artery course in patients undergoing nasal enhancement. They proposed a nasal augmentation method that minimizes vessel damage by confirming blood vessel distribution patterns through ultrasound before injecting soft tissue filler.	Patients had augmentation rhinoplasty using soft tissue filler. Injections of filler were performed under ultrasound examination.	There is a possibility of vascular compromise when injecting the filler through a needle or cannula into the pre-periosteal layer. The safest approach may be injecting the filler into the preperiosteal layer using a large cannula.	[52]/2019
Patients with nasal bone fractures who received rhinoplasty and fracture reduction concomitantly were examined for surgical techniques and outcomes.	Three major types of nasal bone fractures can be distinguished based on computed tomography and preoperative facial images. Two otolaryngologists evaluated The surgical outcomes independently after a telephone survey was conducted to assess patients' satisfaction.	In acute nasal bone fracture cases, concomitant rhinoplasty with fracture reduction may result in better aesthetic results.	[53]/2018
In this study, the authors sought to determine if osteotomy was available and safe for East Asian patients undergoing esthetic rhinoplasty.	The retrospective chart review was done for 227 patients who had undergone nasal osteotomies and silicone implants. Rhinoplasty Outcome Evaluation tested patient satisfaction after surgery. A cadaveric study also involved an osteotomy on each side of the nose on 5 fresh cadavers.	Percutaneous lateral osteotomies and paramedian oblique osteotomies effectively reduce broad nasal bones, thus allowing for reliable augmentation with silicone.	[54]/2018
The objective is to develop tools that can be used to objectively evaluate the necessary dimensions for a customized-glue-diced cartilage construct for dorsal augmentation.	Researchers used the ACAS to modify diced cartilage glue.	Compared to diced cartilage glue, this technique is superior. Alloplastic implants look similar to the shape with varying heights and widths. For a more natural look, the cephalic and caudal ends taper. Improved aesthetic lines at the brow tip.	[55]/2018

#### Bone

Cortical bone grafts are also an option for rhinoplasty. The most common donor sites are the skull, ribs, and iliac crests. For traumatic midface injuries and hypertelorism reconstructions, the skull presents an ideal donor site since it is adjacent to the operative site. For isolated rhinoplasty procedures, other donor sites can be considered. A nasal deformity that cannot be adequately corrected with other graft materials may benefit from bone grafts, as they provide excellent strength and stability. It is very stable and reliable to use bone grafts over a long period [1]. However, because the bone is rigid, it is difficult to shape intraoperatively, requiring saws, burrs, and drills; these tools can create heat that kills osteocytes and denatures growth factors, resulting in graft resorption. In contrast with endochondral bone from other donor sites, such as the ribs and the iliac crest, membranous bone from the skull may not resorb as quickly as endochondral bone from other donor sites. Furthermore, cranial bones can be used because of their proximity to the operation field, and their scarring can be concealed within a hair-bearing scalp [1, 21]. The harvesting of cranial bone grafts can cause alopecia, as well as defects of total thickness, intracranial hemorrhage, and brain injuries. Split cranial bone grafts can be produced in situ or ex vivo from a full-thickness craniotomy segment. As a result of the potential morbidities associated with the donor site, this graft is best suited to surgeons performing craniofacial reconstruction routinely. This approach can be used for midline nasofrontal reconstructions involving craniofacial approaches and possible craniotomies [1].

According to Cizer et al. (2022), inferior turbinate bone grafts are commonly used in rhinoplasty to increase the width of the nose. They approached the lower turbinates in the nose’s lateral wall with an erectile surgical approach. The nasal mucosa has a multilayer squamous epithelium surrounding the middle spongy bone. It is necessary to perform rhinoplasty with a medial flap to harvest inferior turbinate bone grafts. In the inferior turbinate, lidocaine and adrenaline solution are infiltrated after decongestion. Superior and anterior margins of the inferior turbinates are incised mucosally. Medial mucosa elevates along the lower turbinate from its beginning until its horizontal part. Using Heymann nasal scissors, the lower turbinate and lateral mucosa are excised. The excision is completed by directing the scissors inferiorly before reaching the posterior attachment point of the lower turbinate. From the periosteum, the inferior turbinate’s lateral mucosa is peeled extracorporeally. The inferior concha bone is crushed with a straight Kelly clamp to achieve the desired shape. With scissors, people can make the necessary corrections again. As soon as the septum and dorsum are completed, the prepared ITBG is positioned in the planned area. A saddle nose deformity is caused by insufficient septum cartilage in trauma and granulomatous diseases. Thus, ITBG is designed primarily for the reconstruction of saddle nose deformities. Similar to septal cartilage grafts, nasal DA also has increased indications. There have also been successful results using this technique for minor dorsal deficits. During IT, the insertion should be advanced inferiorly so as not to damage this branch. Some cases require postoperative nasal packing or cauterization. It is typically detected and controlled intraoperatively. In addition to mucous cysts, turbinate bone mucosa can cause a mucous cyst in the nasal dorsum. A mucous cyst that has developed requires total excision. Apart from the postoperative care and applications for septorhinoplasty, there is no need for special care or precaution for patients undergoing ITBG. Four years of follow-up revealed no evidence of graft resorption [13].

The study by Mehta et al. (2021) discusses their experience using olecranon bone grafts in severe saddle nose deformities. Twelve patients underwent olecranon bone grafting on their dorsal nasal augmentations between 2011 and 2020. During the surgery, grafts were inserted through old nasal dorsum scars in all ten patients with congenital deformities. At the nasion, in both cases, the grafts were fixed with screws. X-rays of the nasal bone and clinical photographs were used to assess each patient's graft resorption postoperatively. A nasal DA with Olecranon bone graft exhibits minimal donor area morbidity and simplifies harvesting. The Olecranon cortex should be thick enough to allow custom molding and resist resorption over time, thus providing a desirable outcome [56].

An evaluation of freeze-dried cortical allograft bone for nasal dorsal augmentation was performed by Clark et al. (2019). This is an analysis of 62 patients over 10 years following the 42 months. Sixteen out of 19 allografts showed objective evidence of continued volume or neovascularization. According to the patients’ subjective opinion, 37 out of 43 grafts were reported to have had volume persistence. An overall success rate of 85% was achieved. Allograft bone made from freeze-dried allografts can be used safely and effectively as a substitute for human bone donated. Whether allograft bone can be partially demineralized so that it can be carved with a scalpel can be confirmed in future studies for young patients needing long-term reconstruction [57].

#### Cartilage

A good graft material for dorsal augmentation is autologous cartilage. A variety of cartilage contains autologous tissue, including conchal bowls, nasal septums, lateral crura, and costa. Biocompatible materials can be used to wrap cartilage grafts with Surgicel. Due to its original publication, medical cartilage wrapped in Surgicel is also called a Turkish delicacy. The most common complications with autologous cartilage include graft resorption, infection, and implant migration. In the case of revision or secondary rhinoplasty without autogenous cartilage, special care must be taken [31, 58, 59]. Studies assessed the effects of secondary rhinoplasty on patients with cleft lip and palate. An onlay tip graft, columellar strut implant or graft, and lower lateral cartilage repositioning were all included in rhinoplasty procedures. The septum and concha were harvested for cartilage, while the ribs were not used. As part of the research, the Rhinoplasty Outcome Evaluation (ROE) survey was administered, the Mortier scoring scale was modified, and 8 intranasal symmetry measurements and 4 nasofacial measurements were measured. A comparison was made between preoperative and postoperative patient satisfaction and nasal esthetics [41, 60–63].

##### Auricular cartilage

Near the nose, the ear is a readily available cartilage donor site. This graft is considered viable since it is nearby and easy to harvest. Both ears can be harvested to get enough cartilage, and septal cartilage can be preserved when auricular cartilage is used. Unlike secondary donor sites, there is no need to include the ear in the primary site. The conchal bowl can be harvested anteriorly and posteriorly. The nose’s dorsum cartilage can be augmented by 3 to 6 mm. The best results are not always possible when handling auricular cartilage. The natural curvature of auricular cartilage makes dorsal nasal profiles difficult. This material is brittle, elastomeric, and lacks rigidity in structural applications. Furthermore, scar contractions can occur due to auricular cartilage's inherent memory and propensity for resorption [1]. Various reconstructive procedures, such as aesthetic rhinoplasty, can benefit from Auricular cartilage grafts, according to Zinser et al. (2013). This study evaluated the clinical viability, indications, and morbidity of tragal cartilage and auricular cartilage from concha and scapha in rhinoplasty. There were 170 grafts in total between 150 augmentation rhinoplasties. Trachus, concha, and scapha were the sites of donor tissue. Pearson's intraclass correlation was used to assess intraobserver reliability. Scapha harvesting time averaged 27 (8) min, tragus harvesting time 4.5 (1.4) min, and concha harvesting time 5.7 (1.6) min. In order of size, the concha, tragus, and scapha provided the largest grafts. Tips, columella struts, shields, rims, and dorsal onlays were all grafted [64].

##### Costal cartilage

Dermofat grafts absorb more quickly than nasal cartilage and provide significant volume. Carving and placing the graft requires high skill, even though harvesting the cartilage can result in pneumothorax and chest wall scarring. Dermofat or diced cartilage can produce a much better shape with a better definition and predictability when performed by an experienced surgeon. Compared to septal extension grafts, dorsal augmentation requires more costal cartilage. Because it is longer and straighter, the seventh rib cartilage is ideal for augmenting the dorsal area. There are inframammary folds that prevent access to the seventh rib cartilage. Costal cartilage must be carved for DA. Perfect fit requires precise carving. The graft will develop deviations if the operator cannot carve it to the correct dimensions and shape. DA is required for most Asian patients with low dorsums. Warping of the graft is minimized by carving the core cartilage concentrically [5, 65, 66]. As part of their research, Lee et al. (2021) reviewed all patients who underwent rhinoplasty with crushed autologous costal cartilage used for dorsal augmentation. A photo before and after three facial plastic surgeons used the procedure to evaluate its success. The level of satisfaction of patients was also assessed. Postoperative complications were investigated, as well as anthropometric measurements. 39.5, 47.45, 13.1, and 0.0% of facial plastic surgeons' evaluations were excellent, good, fair, and poor results. In 42.1, 39.5, 15.8, and 2.6% of cases, patients were delighted, satisfied, moderate, and dissatisfied. In the postoperative period, there was a noticeable increase in the heights of the dorsum and radix. Dorsum complications included 28.9%, surface irregularities, five resorptions, and two short noses. Crushed autologous costal cartilage can significantly increase the nasal dorsum. When surgery is indicated, this technique may be a better alternative due to the high rate of complications at the recipient site [30]. In a study by Toriumi et al. (2017), autologous costal cartilage or micro fats were used to augment the dorsum with autologous cartilage. With the increasing demand for augmentation rhinoplasties and the increased use of graft materials for structural rhinoplasties, costal cartilage grafts are becoming more popular. In dorsal grafting, costal cartilage is an art and a science. Dorsal grafts are carved to match the contour of the nasal dorsum. A contoured graft must be fixed to the bony dorsum and contoured according to the patient's desires to minimize the risk of warping and displacement. There is a discussion of the different types of dorsal grafts and how they are fixed. Dorsal grafts are fused to the perforated or rasped nasal dorsum through the perichondrial interface. This creates a natural-looking and immobile nasal dorsum. Fixing the dorsal graft decreases warping and deformity. In rhinoplasty, microfat could camouflage compromised tissues and promote healing. A surgeon can enhance soft tissues by injecting micro-fat into an open surgical field, promoting healing and enhancing healing effects using MISTA. After injection into soft tissue carriers, a microfat implant is placed into the surgical field. The use of MISTA in cosmetic and reconstructive surgery is tremendously beneficial [6].

An article by Yoo et al. (2019) discusses conventional and recent applications of costal cartilage in Asian rhinoplasty. Costal cartilage has been used for Asian rhinoplasty techniques in different studies. There are two types of costal cartilage commonly used for dorsal augmentation: solid-block cartilage and diced cartilage. Surgeons have reported numerous types of grafting techniques for tip and septum reconstruction. Surgeons should consider both donor-site morbidity and graft complications when performing costal cartilage grafts. Several strategies have recently improved the prevention of these complications [67]. Warping after ACC implants is the most common complication reported by Miranda et al. (2013). Three patients received monoblock ACC implants, and one received laminated ACC implants in the present patient group. The second most common complication was resorption in the ACC group. All four patients who had resorption received monoblock augmentation in this study. Surgeons should remember that resorption can occur with any dorsal costal cartilage implant [30]. According to Yilmaz et al., previously, ACC was used for dorsal enhancement without high resorption rates, and the shape of the nose was satisfactory. Future studies are needed to clarify this discrepancy [3].

A study by Ujamet et al. (2123) concluded that autologous costal cartilage was an excellent graft material for nasal reconstruction as it provided both volume and quality. Most commonly, costal cartilages from the 5th- 8th ribs are harvested for augmentation grafts. Despite this, the 10th rib offers excellent graft potential and is often overlooked. Using rib cartilage to repair secondary cleft nasal deformities has rarely been documented in the literature. Their simple and rapid technique makes rib cartilage worthwhile for secondary rhinoplasty and nasal deformities caused by cleft lips. Researchers have demonstrated the ease of harvesting, the stability, and the predictability of outcomes using the 10th rib cartilage in several clinical cases. Various challenging secondary cleft rhinoplasty procedures with excellent results have been successfully performed with the 10th rib graft by the senior author. Several advantages make the 10th rib graft a good option for such procedures, including its easy harvestability, dynamic cartilage properties, and predicted outcomes [68]. Using autogenous cartilage grafts to enhance rhinoplasty, Leach et al. (2018) reported long-term patient-reported outcomes after open augmentation rhinoplasty with autogenous cartilage grafts. An overview of operative steps and perioperative care was provided to optimize results. From 2008 to 2016, 11 such augmentation rhinoplasties were reviewed retrospectively. An advanced cosmetic westernization of the nose was indicated in cases of saddle nose deformity, the post-traumatic collapse of the nose, and post-traumatic nasal deformity. In this study, a patient questionnaire was used to measure the long-term outcomes as reported by the patient. Nose shape, position, and function improved significantly in all patients. As a result, there were no cartilage exposures, warpings, or resorptions and no recurring deformities. A second correction was successful with the above technique for a patient whose dorsal graft fractured 2 years later. It took 5.2 years on average for the study to be completed. In the follow-up questionnaire, 100% of nine patients expressed satisfaction with the appearance of their noses. No nose problems were reported by 100% of respondents during follow-up. A low-volume operator successfully performed augmentation rhinoplasty with L-shaped costal cartilage grafts for diverse indications. This technique can provide long-term patient satisfaction and pleasing aesthetic results [69].

##### Septal cartilage

Dorsal augmentation is often performed with septal cartilage. Due to its rigidity and flat, planar geometry, this graft is easily carved into any other nasal tip graft. There is a limited supply of septal cartilage. With smaller noses, surgeons have less septal cartilage available for DA procedures. A traumatic injury or revision rhinoplasty that already damaged septal cartilage makes the situation worse. Slightly deviated septums can also make harvesting grafts difficult. Over-resection of the septum can lead to septal perforations, and not considering the septal lining can compromise the structural integrity of the nose. In sufficient quantities, septal cartilage remains popular as a graft [1]. CCDG grafts were evaluated by Lee et al. (2022) for their postoperative feasibility. A retrospective review of 38 rhinoplasty records of patients who underwent an open approach to DA was conducted. Twenty patients (52.6%) received CCDG graft compared to 18 with DG graft (47.4%). A study comparing anthropometric data from facial photographs with satisfaction questionnaires analyzed aesthetic outcomes and palpable irregularities on the nasal dorsum after surgery. There was a successful outcome for both groups in terms of aesthetics. After surgery, there was a more significant increase in dorsal height, radix height, and tip projection compared to preoperative data. Rotation of the tip was not significantly increased. Both groups had significant palpable irregularities, although aesthetic satisfaction was similar. A significant difference was seen between the CCDG graft group and the DG graft group regarding dorsal irregularities. Furthermore, two surgeons evaluated the CCDG graft group significantly better than the DG graft group regarding the degree of irregularity. A revision rhinoplasty was not performed on any of the patients who complained about an irregular dorsum. An alternative to irregular dorsum complications can be achieved with CCDG grafts [70].

**Fig. 1:**
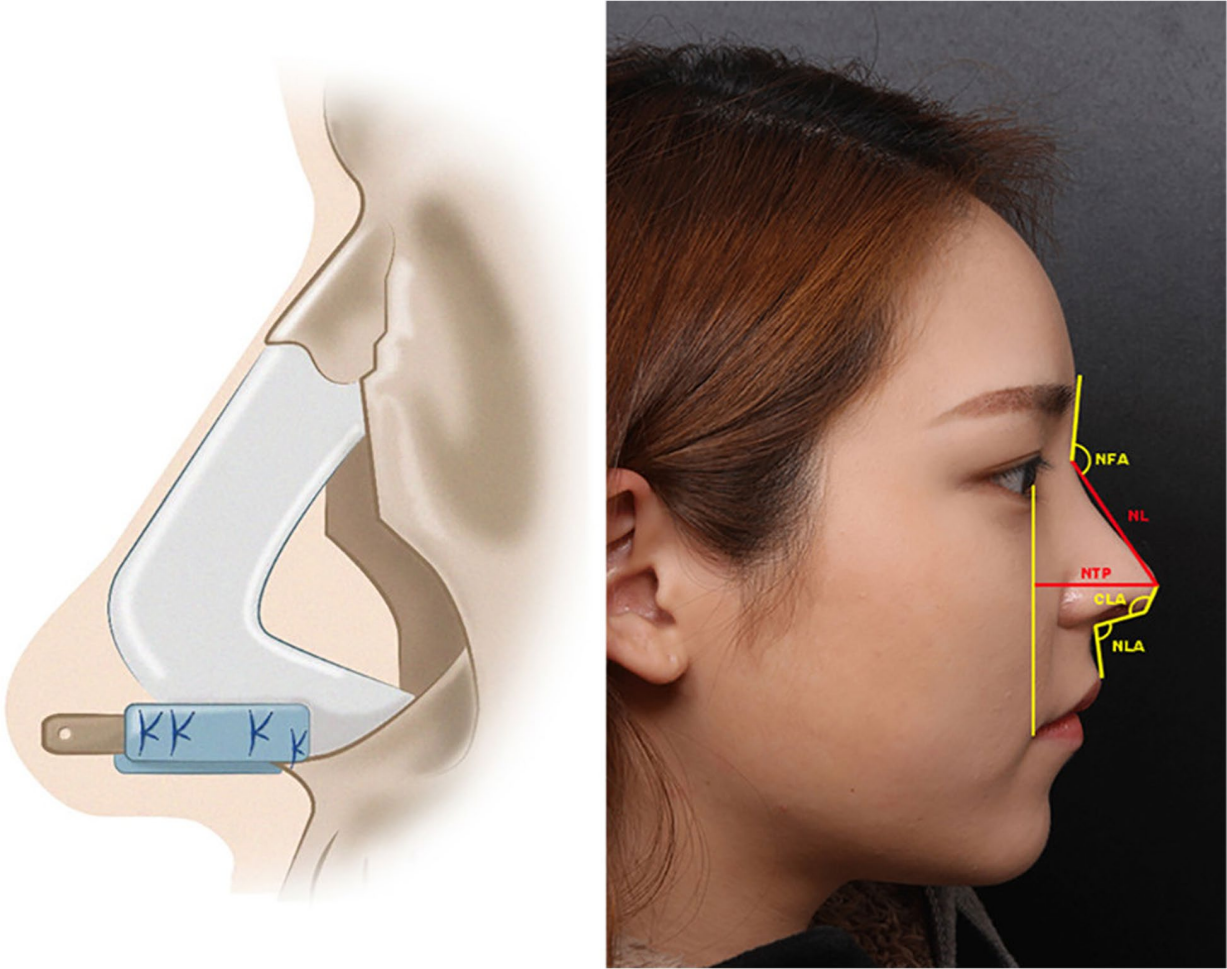
Techniques of surgery and anthropometric measurements. An illustration of a bone and cartilage complex (left). NLA, nasolabial angle; NTP,nasal tip projection; NL, indicates nasal length; CLA, columella-lobular angle; NFA, nasofrontal angle. Reprinted from ref [72] (Open Access Contents)

**Fig. 2:**
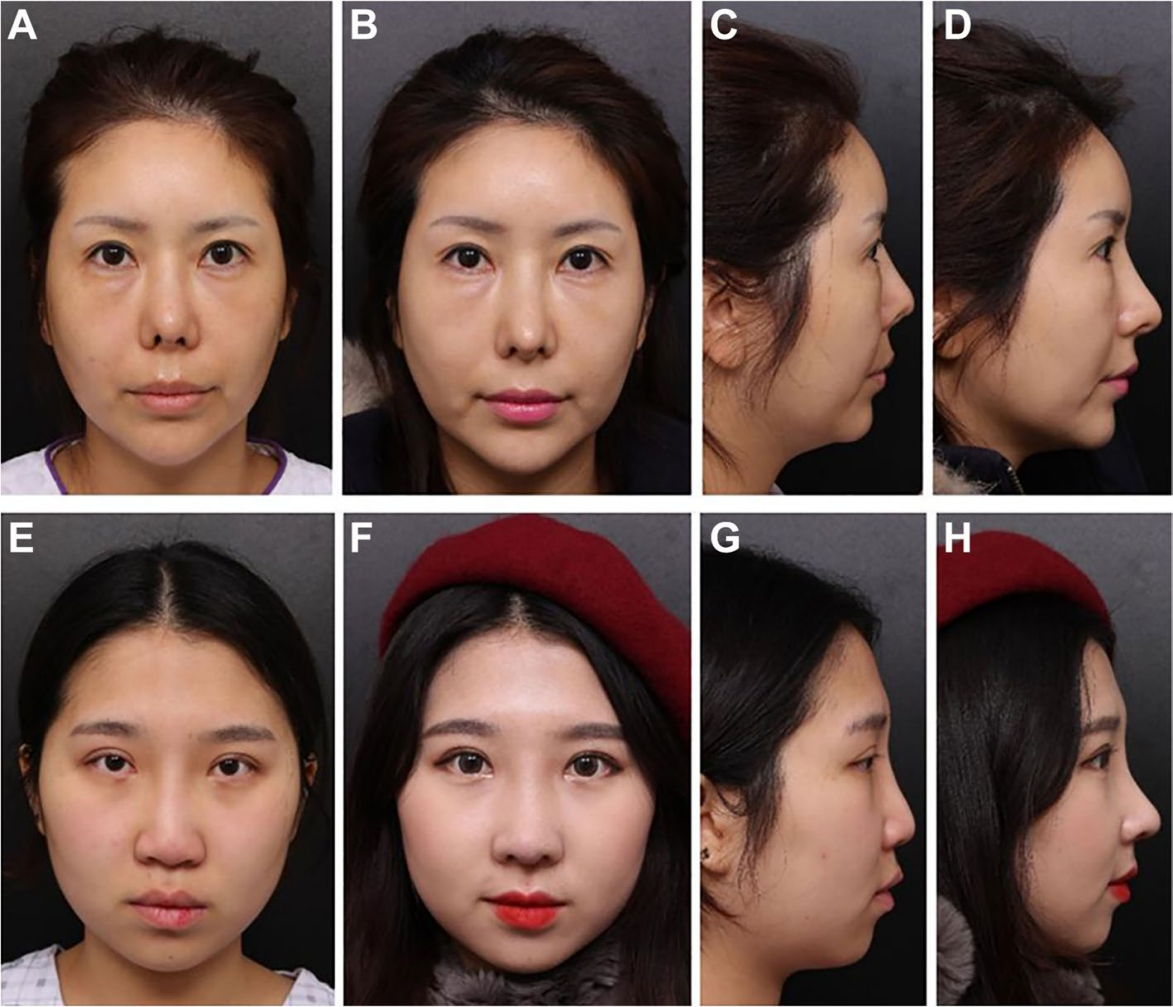
**A** and **C**, **E**, and **G** Photographs of the face taken before surgery. **B**, **D** 10 months. **F**, **H** 12 months after surgery. Reprinted from ref [72] (Open Access Contents)

Lee et al. (2016) defined a septal L-strut to prevent the collapse of the nasal dorsum as pressure moves from the rhinion to the supratip breakpoint. Using computed tomography, a model of the L-strut with a width of 1 cm was developed. If the superior L-strut is to be preserved, at least 45% of it must be preserved. To prevent collapse or distortion of the caudal L-strut, augmentation rhinoplasty must preserve or reinforce it. As the DA material moves toward the supratip breakpoint, an augmentation pocket must hold it. To better understand why target tissues deform and collapse, clinicians used a numerical analysis utilizing a FEM-based model [71]. Ahn et al. (2019) introduced a new septal extension graft using a cartilage-bone complex and cartilage. An external approach and sedative anesthesia were used for all operations. The perpendicular plate of the ethmoid bone and septal cartilage were harvested through septoplasty. A “sandwich technique” differs from other SEGs14 by using linked septal cartilage and bone instead of inserted bone between layers of cartilage (Fig. 1). A complex cartilage-bone projection and lengthening was created by two layers of cartilage on each side. Creating strong fixation, the double-layered cartilage portion of the L-strut was placed on the caudal septum of the L-strut and the bony portion on the tip side. During septorhinoplasty, 30 consecutive patients were treated. All procedures were performed using a sandwich technique that combined cartilage with bone. External approaches were used in all cases, and septoturbinoplasty was also performed. Additionally, 21 osteotomies, 10 augmentations using silicon, 10 augmentations using Alloderm, and 7 paramaxillary augmentations were performed with tip plasty. The surgery results were satisfactory for all patients (Fig. 2). According to specialist opinions, there were three excellent and 23 good cases. There were 26 successful surgery outcomes, including excellent and good outcomes. No case was poor, and four cases were fair. Twenty-four cases resulted in no complications, three resulted in notable transcolumellar scarring, three resulted in mild nostril asymmetry, and one resulted in cartilage gauziness. As of the study’s follow-up period, there were no cases of infection, nor were there any cases requiring reoperation. Using cartilage and bone complex, the sandwich technique can be very effective for Asians with weak nasal tips [72].

##### Ear cartilage

Ear cartilage is often used in rhinoplasty. Different methods have been used to harvest, prepare, and apply conchal cartilage grafts. It is readily available and accessible. Due to its elasticity and intrinsic curvature, ear cartilage is unsuitable for weight bearing. As a result, conchal cartilage is commonly used as a stacked or fascia-wrapped DA graft. Conchal cartilage diced with attached perichondrium is suitable for patients with moderate to thick skin and little risk of infection. Segmental dorsal grafts can enhance a mildly flat nasal dorsum or repair a sunken supratip. A large piece of conchal cartilage must be harvested for a full-length nasal augmentation. Convex shapes can be cut from large pieces and stacked to eliminate their natural curve. Long-term DA using such methods may leave the graft visible. It is not recommended to use conchal cartilage for severe dorsal deficiency [34].

##### Conchal cartilage

Secondary or reconstructive rhinoplasty can benefit from conchal cartilage grafts because they are easy to harvest and produce good results in the long run. The conchal cartilage has several anatomical characteristics, and the harvest technique is well-documented. Conchal cartilage’s intrinsic curvature makes it a poor material for DA due to its inability to produce symmetrical results. Additionally, the conchal cartilage rarely yields a piece large enough for a single piece of dorsal augmentation. Conchal cartilage has an intrinsic curvature, which can be overcome by multilaminar cartilage. Conchal cartilage is intrinsically irregular but becomes apparent after surgery. This problem has also been avoided using conchal cartilage divided and wrapped in fascia, which has gained widespread acceptance as an ideal method for DA [73]. With conchal cartilage, it is possible to augment the nasal tip similarly to LLC. Due to its inherent curvature and limitations in length, conchal cartilage has limited usage as a DA material. A large amount of cartilage must be harvested to augment the nasal tip and radix with conchal cartilage. Stacking multiple layers of a convex shape eliminates its natural curvature. The graft will be visible over time when DA is performed through such methods. DA often uses wrapped diced conchal cartilage or conchal cartilage. Hematoma and keloid formation are likely at the donor site when using conchal cartilage for substantial augmentation. The recipient may also develop irregularities in their dorsal surface, graft resorption, and infection [74].

Using retroauricular fascia and conchal cartilage, Varedi and Bohluli (2015) developed a composite graft. For dorsal augmentation, especially segmental augmentation, diced conchal cartilage compared well to intact perichondrium, according to the senior author of this paper [75]. The augmentation of the dorsal region with diced conchal cartilage wrapped in Surgicel was reported in Erol (2000) [76]. Dorsal enhancement can also be achieved quickly and conveniently with diced cartilage and warm blood, as Codazzi and colleagues (2014) reported [77]. By using diced cartilage grafts wrapped in temporalis fascia, Daniel (2008) modified its application. Because this procedure is easy to prepare and results in less resorption, it has gained popularity over the past decade [78]. A fibrin glue graft with diced cartilage provides favorable aesthetic results and a low resorption rate, according to Tasman (2015) [79].

Varedi et al. (2015) presented their experiences with augmentation of the dorsum using conchal cartilage and retroauricular fascia. Study participants had moderate to severe dorsal deficiencies. An auricular cartilage and postauricular fascia biopsy was performed through the postauricular sulcus. A layer of conchal cartilage was used if there was a moderate amount of dorsal deficiency. More severe deficiencies required a multilayer conchal graft, which superimposed cartilage pieces. 6-0 polydioxanone (PDS) sutures were used to attach cartilage segments. The cartilage segment was then fixed to the postauricular fascia with 6-0 PDS sutures. It was necessary to undermine the nasal dorsum to place chondrofascial grafts carefully. Following functional disorders, chondrofascial grafts were inserted in the dorsal pocket after tip plasty and lateral wall osteotomies. This study included fourteen participants. It took an average of 25.4 months to complete the follow-up. There was a wide range of ages among the patients. The grafts were not reabsorbed, displaced, or asymmetrically reabsorbed in the second week. Neither the donor site nor the postoperative scar was visible. The donor sites were affected by mild ecchymosis in eight patients. A small amount of graft resorption was observed in three male patients. Performing revision surgery on these patients was unnecessary because the esthetic results were not compromised. Composite turbinate cartilage and retroauricular fascia grafts were effective in patients with moderate to severe nasal dorsal deficiency [80]. Kim et al. (2015) studied diced conchal cartilage attached to the perichondrium to augment nasal dorsums, radixes, and tips. The records of 37 patients with diced conchal cartilage and perichondrial attachments inserted into the nasal dorsum, radix, or tip were reviewed. Face-to-face comparisons of pre- and post-operative photographs were used to assess postoperative surgical outcomes. The analysis also included complications. A valuable graft material in rhinoplasties is diced conchal cartilage with perichondrial attachment [81].

##### Diced cartilage with fascia graft

Costal cartilage provides enough cartilaginous material to augment the low dorsum of Asian patients, making it the best cartilage graft for significant dorsal augmentations. However, solid block costal cartilage grafts can warp and show through thin skin. In addition to wrapping diced cartilage in the temporal fascia, diced cartilage can also be used to overcome such drawbacks. Silicone implants have replaced diced cartilage grafts that were used decades ago. The researchers, however, found the graft undergoes total resorption and attributed the process to foreign bodies reacting with Surgicell. Their report describes a modified method of wrapping diced cartilage in temporal fascia. It is easy to perform and results in a lower resorption rate, making the method readily adopted by numerous surgeons. Diced cartilage grafting effectively removes dorsal irregularities or enhances the dorsal area [5]. Diced cartilage grafts are unpredictable regarding resorption, which can result in overcorrection and under-correction. The extent of augmentation provided by diced cartilage grafts is limited, which prevents them from providing structural nasal support. Larger grafts have a greater migration risk, whether the entire graft or the diced cartilage itself, which can result in palpable and visual irregularities. Diced cartilage can also result in “amorphous” aesthetic lines due to its nature, unlike more rigid alternatives that produce more precise contours due to its rigidity. Several reports have described dermal scarring that leaves the overlying skin looking like cobblestones, especially in thin-skinned patients. For those who require moderate amounts of augmentation between 1 and 4 mm, diced cartilage does present a viable option for DA [1]. An easier way of using GDCG was developed by Yoo et al. (2022). A novel mold for DA is described here about GDCG. DA with molded GDCG was used in the study on 80 patients. Reviewing facial photographs and medical records assessed postoperative complications and patient satisfaction. To examine changes in implant widths and heights over time for 23 patients who underwent three-dimensional scanned imaging, dorsal widths and heights were measured at the radixes and rhinions. After 3 months, the latest follow-up photograph was compared with the 3-month postoperative picture to assess graft resorption. Three-dimensional scanned images were used to assess the change of the dorsum over time. Surgery was found to be successful for 66 of the patients. Nineteen patients experienced complications, and 8 had to have their procedures revised. As a result of the resorption of the GDCG, one patient underwent revision surgery. Comparing preoperative and 1-year postoperative data, 3D scanning showed increased dorsal height without an increase in dorsal width. A 3-month postoperative dorsal height comparison to a 1-year postoperative dorsal height showed no significant progress. Based on their findings, gluing diced cartilage to a unique mold for augmentation in the dorsal region could be helpful for rhinoplasty [33].

According to El Abany et al. (2023), diced cartilage grafts can enhance the dorsal part of the nose during rhinoplasty. From 2017 to 2021, rhinoplasty with dough grafts was performed on patients. SCHNOS-C and scale of visual analog score completed preoperatively and postoperatively are required. There were two postoperative periods: 6 months and more than 6 months. Postoperative scores were compared using Wilcoxon rank-sum tests and paired *t*-tests, with preoperative scores compared with preoperative scores. DA with diced cartilage grafts is an effective technique for improving the dorsal aesthetics [63].

In primary and revision rhinoplasty, Hudise et al. (2021) used TF grafting to repair the nasal dorsum, hide nasal irregularities, and improve nasal contouring. TF in tubed form was used to contour the nasal dorsal area. Patients were asked about their satisfaction with contour definition, dorsal irregularity, and dorsal irregularity. There was a correlation between tubed TF used for DA and postoperative outcomes. Rhinoplasty Outcome Evaluation questionnaires were used to assess patient satisfaction. An independent rhinoplasty specialist examined and palpated the dorsum of the nose to evaluate the DA results. The tubed TF was administered to 74 patients. Most often, TF was used to treat thin skin. Upon inspection and palpation, no irregularities were found in the graft. There were no complications at the reception site. During the donation procedure, one patient developed a mild hematoma. Postoperatively, the mean patient satisfaction score was 19.95 compared to 10.14 preoperatively. Additionally, the tubed TF accentuates dorsal contours and improves definition, covering all irregularities [82].

In their comprehensive review of diced cartilage techniques in the databases, Dong et al. (2022) emphasize using different wrapping materials. There is a possibility that free diced cartilage may gather in certain areas and cause irregularities postoperatively. Due to severe reactions to foreign bodies, Surgicel isn't used as often as other non-blood wrapping materials. Donor site morbidity, insufficient quantity, and long recovery times are the apparent disadvantages of fascia. Tutoplast-treated fascia lata, AlloDerm, and esterified HA wraps for cartilage have produced encouraging initial results, but their use has been controversial. Wrapping shaved cartilage or ultra-diced cartilage in concentrated growth factor or platelet-rich fibrin gave satisfactory clinical results [83].

According to Beaudoin et al. (2023), dorsal irregularity is among the most common rhinoplasty problems. A rhinoplasty surgeon recommends graftings along the hole dorsum to achieve a natural, unoperated appearance. Choukroun’s platelet-rich fibrin scaffold includes diced cartilage among its most recent innovations. It is unknown if this technique can provide millimetric precision when creating thin, malleable, and reproductive grafts. The senior author has developed a new template to allow the creation of standardized-sized and thickness reproductive grafts [84, 85]. Patients who underwent a millimetric DA with iPRF or a PRF and utilized diced cartilage were evaluated at least 6 months after this retrospective case series. His newly developed template operated 54 cases from 2018 to 2022. Using the template, soft grafts made from diced cartilage can be fabricated quickly and reliably [86].

It was presented by Fung et al. (2023) for revision rhinoplasty in patients with complicated silicone augmentation. They used molded GDCG. After removing silicone implants, 28 patients underwent costal cartilage augmentation for dorsal augmentation. Analyzed were demographics, surgical technique, anthropometrics, and complications in patients. An anthropometric measurement and a score for aesthetic outcome were performed. Asians commonly undergo revision rhinoplasty following failed silicone augmentations. An accepted complication rate of 2% is achieved with molded GDCG for revision DA [87].

In Park et al.’s (2016) study examining DCIF’s efficacy in nasal augmentation in Asian patients, the strengths and weaknesses of DCIF were examined in depth. Within the last 2 years, 15 patients for whom DCIF was used for major DA underwent a retrospective review. A deep temporal fascia was wrapped around diced cartilage. The dicing materials in 11 cases were costal cartilage and a mixture of septal and conchal cartilage in four cases. DCIF is typically inserted at the supratip from the radix. Ten out of 15 cases achieved satisfactory aesthetic and functional results; they were followed up for an average of 13.3 months without complications. Five resorption cases were observed at the site of fascial harvesting, including mild deviations, slight supratip depressions, and irregularities of the nasal dorsum. DCIF has been found helpful for augmenting the dorsal portion of the nasal bridge; however, certain complications arise during the procedure. Despite obvious warping being avoided, slight deviations are still possible, requiring further refinement of the technique [88].

#### Fat graft

Fat grafting continues to be a commonly performed procedure despite easy harvesting, abundant graft material, and rare transplant rejections. Structural fat grafting has proven to be an effective treatment strategy thanks to extensive research and refinement of surgical techniques [89]. Open rhinoplasty and multiple revision procedures have resulted in atrophic, damaged, and contracted nasal skin in recent years. A damaged skin envelope poses a significant challenge for tertiary rhinoplasty cases because of these issues. As a result of vascular compromise, injectable fillers can damage nasal skin envelopes. Novel techniques can conceal these irregularities, thicken the nasal skin, and heal the vascularly compromised skin. Skin damage can be camouflaged and restored with autologous fat. Fat cannot be injected quickly into a surgical field in all these cases. I camouflage nasal irregularities using autologous fat and restore atrophic nasal skin [6, 89, 90]. The skin must heal over the nasal supports before or after the nasal supports are rebuilt by injecting autologous fat. Due to the need for open dissection in significant reconstructive surgery, autologous fat cannot be injected into closed soft tissue spaces. Autologous microfat can increase soft tissue by infiltrating the costal perichondrium or temporalis fascia. Consequently, microfat is injected into soft tissue. Due to its highly filtered nature, microfat is easy to inject into the temporalis fascia or costal perichondrium. Augmenting and camouflaging any open spaces left after rhinoplasty dissection with autologous/composite soft tissue grafts [6, 89].

An ampoule containing epinephrine is injected after a solution of 1 L of lactated ringers is injected with 50 mL of lidocaine 1%, and an ampoule containing epinephrine is injected to harvest the microfat. The fat particles are broken up with a cheese grater liposuction cannula before being collected from the patient's flanks or periumbilical region. Fat is separated from the other layers of collected fat after centrifugation for 4 min. A fine filter is then used to filter out the fat layer, leaving a tiny aliquot of ‘microfat” that will be loaded into tuberculin Luer lock syringes of 1 mL in size. A delicate tea strainer can be used to achieve the right consistency of the microfat for filtration. A 27-gauge needle injects the microfat into the interstitial layers of the costal perichondrium or temporalis fascia. As a result of injecting microfat into the temporalis fascia and coastal perichondrium, the coastal perichondrium will be plumped up and filled with fat. A microfat delivery vehicle is a soft tissue that delivers microfat to a specific area of need. A microfat-infused soft tissue envelope is placed over the area below the nasal dorsum or tip to smooth out irregularities or assist in recovering damaged soft tissue envelopes. Care must be taken to prevent pressure on MISTA-treated areas and the graft from becoming distorted. Glasses should not cover this area of the nasal dorsum for six weeks to avoid any injury. A MISTA procedure would be especially beneficial for patients with thin, damaged dorsal nasal skin from rhinoplasty or with long-lasting scarring from previous rhinoplasty. Patients treated with MISTA therapy have significantly improved their skin thickness, turgor, color, and vascularization. Long-term results are better since scar contracture has been dampened [6]. This technique has tremendous potential as a soft tissue augmentation or salvage technique. Additionally, soft tissue augmentation is required for the reconstruction of facial and breast structures, burn management, and recovery from chronic wounds and irradiated areas. In an open surgical field, MISTA is ideal for soft tissue augmentation in situations where microfat is not an option because of the open surgical field. Also, MISTA eliminates the need for fat injections, which can result in unintentional intravascular injections that lead to vascular compromises, skin necrosis, and blindness. Accidental intravascular injections are eliminated by using the soft tissue delivery vehicle. Implanting microfat-infused temporalis fascia into atrophic inferior turbinate tissues may restore the ability of the turbinate tissue to humidify the nose without causing blindness. Repairing wounds and tissue beds more efficiently and precisely with microfat-infused microvascular-free flaps or any other microvascular-free flap would be possible. MISTA has excellent potential to significantly impact many aspects of cosmetic and reconstructive surgery [6]. A growing body of research suggests that periorbital fat grafting must deliver minute amounts of fat into thin skin areas. Due to its relatively thin skin and limited space, the nasal dorsum is a challenging anatomic site for placing delicate fat parcels. It is more likely that larger fat parcels will result in dislodgment, nodulation, and irregularities of the skin following implantation. Studies that use fat grafting for rhinoplasty usually use lipo injections to refine or reconstruct the nose [6, 89, 90].

In Asians with augmentation rhinoplasty performed by Kao et al. (2016), one-third of maneuver MAFT showed long-term success. This retrospective study examined 198 patients who had primary augmentation rhinoplasty with MAFT. Liposuction was used to harvest fat, which was then centrifuged and refined. A MAFT-Gun transplanted minute parcels of purified fat to the nasal dorsum. Using MAFT for primary augmentation rhinoplasty in Asian patients, they demonstrated the suitability of the fat-transfer strategy [89]. Dermofat grafts have been used for saddle nose reconstruction since Jeong et al. (2022). The gluteal sulcus dermofat graft was used to reconstruct a double-layer saddle nose in two patients with type IV deformities. A practical method for correcting saddle nose deformities using a double-layer dermofat graft, performed under local anesthesia and without cartilage, demonstrated excellent results [90].

#### Filler graft

Compared to standard rhinoplasty with allografts and autologous grafts, injectable fillers are less invasive, simpler, more effective, and cheaper. In this procedure, the dorsum and radixes of Asian noses are enhanced. Selection of patients is crucial to successful postoperative results. Injectable fillers are suitable for patients with minor humps, deviations, low nasal dorsums, and high nasal tips. Fillers contain collagen, bovine fat, hyaluronic acid, paraffin, liquid silicone, and calcium hydroxylapatite. Rather than one single-point injection after injection, performing multiple injections along the straight dorsal line is safer. Avascular planes of the nasal dorsum are ideal for injections. The superficial musculoaponeurotic system is typically injected into the periosteum or perichondrium. Postoperative complications of injection rhinoplasty include asymmetry, swelling, erythema, bruising, and asymmetry. Additionally, extreme vascular complications should be considered. Intravascular injections or fillers can cause such complications. Even though there are no definitive preventative measures for filler injection complications, the following instructions will assist the surgeon [74]. HA is a naturally occurring glycosaminoglycan disaccharide that is biodegradable and biocompatible in the nose. HA and CaHA injectable fillers have been used for years to correct tip, dorsum, and columella deformities. A hyaluronidase enzyme breaks down HA, making nose injections easy to revert and without the risk of an allergic reaction. It is recommended that silicone should not be used in nasal injections because granulomas, infection, and other adverse effects are associated with it [91]. It has been practiced off-label for many years to perform “liquid,” “nonsurgical,” or “injection” rhinoplasties. CaHA (Radiesse) and HA (HA) are commonly used. Nonpermanent fillers offer distinct advantages over permanent fillers at this location, including minimal pain, no general anesthesia, a shorter procedure time, faster recovery, and the reversibility of hyaluronic acid-based fillers. The filler can cause tissue ischemia and necrosis if injected directly into end arterioles or compressed. There is a risk of devastating vascular compromise but no severe complications. A popular facial filler used for correcting deformities following rhinoplasty surgery, Radiesse has good results and minimal complications. Increasingly popular are minor nasal augmentations due to their long duration, moldability, elasticity, and high viscosity. Intradermally injected into the nasal region, HA can cause migration and the Tyndall effect, which causes a bluish tint in bright light. First off-label use of liquid silicone in rhinoplasty [92]. Rhinoplasty can conceal nasal irregularities and smooth out asymmetries with HA fillers. There has been some controversy surrounding the use of fillers due to the possibility of severe adverse events [91]. HA fillers are becoming increasingly popular for both primary and revision rhinoplasties because of their minimally invasive nature, rapid recovery, and immediate results [91].

According to Lee et al. (2019), the dorsal nasal artery was assessed in patients who underwent nasal augmentation with soft tissue fillers, and ultrasound was proposed to confirm the pattern of blood vessels during soft tissue filler injections to minimize vessel damage. The filler was injected into the pre-periosteal layer with a needle or cannula, but vascular compromise was possible. It is possible to inject the filler into the pre-periosteal layer using a large-diameter cannula and a gentle injection technique [52]. Hyaluronic acid (HA) injections into the nose are described by Rauso et al. (2020) to yield pleasing, stable results. The non-invasive rhinoplasty using HA filler was performed on 148 patients. In these cases, the procedure would be contraindicated by anatomic contraindications, including an excessive nasal deviation or a prominent dorsal hump. The surgical plan was the same as that for placing cartilage grafts but with HA injections placed in a specific order to reshape and stabilize the nose. A visual analog scale measured the satisfaction of patients with their surgical results. All patients experienced transient redness and slight swelling after the procedure, but these symptoms disappeared within 24 h. The use of hyaluronidase was used on one patient who developed vascular impairment. In this study, the authors found that HA injections can reshape the nasal bridge swiftly, safely, and effectively with minimal discomfort for the patient. To achieve the best results, it is essential to select patients whose condition is suitable for the procedure and to plan it meticulously before it [93].

#### Turkish delight

Various techniques and materials have been used to augment the nasal dorsum. The most common graft material is autologous cartilage, which remains the gold standard. Using diced cartilage wrapped in oxidized cellulose and named the Turkish Delight technique, Erol described the use of diced cartilage and then wrapped in oxidized cellulose. This technique has been modified to extend its applicability to more complex cases and enhance long-term results [76, 94]. After tissue resolution is complete, it is possible to see carved or crushed cartilage through the nasal skin. Smoother surfaces were achieved by wrapping diced cartilage in Surgicel. One hundred sixty-five patients have been treated for traumatic nasal deformities using this innovative technique; 350 have been treated for facial deformities after rhinoplasty, and 1850 have been treated for primary rhinoplasty using this technique. As part of the surgical procedure, cartilage is broken into pieces between 0.5 and 1 mm in size using a no. 11 blade. The fine-textured mass is infected after applying an antibiotic (rifamycin) and wrapping the cartilage in Surgicel. The dorsal nasal skin is molded into a cylindrical graft and inserted under it. A nasal tip or lateral wall may need to be overcorrected depending on the type of deformity. Stitching the mucosa under the dorsal skin before applying the graft is necessary. The same techniques can augment the nasal dorsum as mild-to-moderate nasal depressions. Costal cartilage can correct the columella and the nose's length for more severe defects. Recurrently, deviating nasal bridges could be treated with this simple procedure. Following the removal of the dorsal portion of the septal cartilage and reattaching the cartilage to the dorsal surface, Surgicel-wrapped diced cartilage was inserted in all patients. When the nasal skin is thin or hump removal is excessive, as well as after hump removal, this technique can effectively camouflage irregularities in the bones. As a complication, swelling increased in six patients following surgery. Among 16 patients, fibrosis persists in overcorrecting, while 11 patients experience excessive resorption. The histology of 16 patients after touch-up surgery was evaluated by removing thin layers of excess cartilage from the dorsum of the nose at 3, 6, and 12 months after surgery. Fibrious tissue connections connect the graft cartilage fragments in a mosaic pattern, as shown in this image. People can obtain a smooth, long-lasting surface when the plasticine-like material is molded with fingers. Despite its favorable results, surgeons routinely use surgically wrapped diced cartilage during rhinoplasty procedures [76]. By encasing small hydroxyapatite-calcium carbonate granules in layers of oxidized cellulose and attaching them, Kurtzman et al. (2017) modified the Turkish Delight technique used for dorsal enhancement and compared its effectiveness with others. Stable clinical results were achieved in each of the four cases examined. The graft remained stable 2 years after surgery using cone-beam computerized tomography (CBCT). In the treatment of nasal dorsum deficiency, hydroxyapatite granules have the potential to provide a valuable alternative to Turkish Delight [94].

#### Acellular dermal matrix (ADM)

ADM is a biocompatible, non-immunogenic material that can achieve DA. ADM is a biocompatible, non-immunogenic material that can achieve DA. Epidermis and dermal components that may cause immune reactions are removed before freeze-drying. The main components of ADM are collagen and elastin, which provide elasticity, strength, and elasticity. As a result, ADM is highly biocompatible. Because the natural dermal structure is maintained in three dimensions, fibroblasts can penetrate, and neovascularization occurs, promoting tissue regeneration [24, 95]. Head and neck surgeries increasingly use ADM for cosmetic and reconstructive purposes. Extraoral and intraoral defects, as well as oropharyngeal defects, can be treated with it. The material can also repair tympanic membranes, nasal soft tissues, and skeletal support [96]. The thickness of implanted ADM does not decrease over time despite histological studies showing increased extracellular matrix protein expression, dense collagen and elastin fibers, and microvessel formation. This allows adequate tissue ingrowth and prevents displacement and exposure for a long time. The most commonly used matrixes are MegaDerm and AlloDerm. MegaDerm is cross-linked and irradiated with e-beams. Cross-linking is responsible for collagen's strength and durability. MegaDerm maintains its rigidity and shape despite reduced resorption and improved fibroblast infiltration [24].

An open or endonasal approach can be used to place ADM. As the flap over the nasal dorsum should be elevated after dissection of the upper and lower lateral cartilage perichondrium, the midline between the nasal dorsum and the new rhinion can guide the placement of the ADM. Upper lateral cartilage and the nasal bones are connected by a subperiosteal plane formed by the periosteum overlying the nasal bone. If this subperiosteal plane is maintained, the ADM cannot shift and become visible after surgery. To reach the nose, dissection continues from the dorsum of the nasal dorsum. When selecting the type of ADM product, it is essential to consider how long the product will be and how thick it will be based on the patient's dorsal height. Place the triangular part of the ADM towards the cephalic part while holding the ADM gently with bayonet forceps. It is essential to check how long and thick the ADM should be over the nasal dorsum. A 15th blade may be used when additional trimming is needed. Indirect vision is used to gently place the ADM in the pocket of the nasal dorsum. Skin closure is performed after final confirmation of the nasal dorsum's size and shape. The majority of ADMs do not require suture fixation. Depending on the situation, it may be necessary to suture the ADM to surrounding structures. The ADM may also be fixed to the outside of the nose using the skin for a week. This alloplastic implant may also be combined with other alloplastic implants due to its low rigidity and absorption potential. ADM and alloplastic implants help treat saddle noses with severe structural problems. As long as the silicone or expanded polytetrafluoroethylene is easily anchored in the surrounding tissues, it can be fixed without displacement into the dorsum without displacement [24].

An augmentation rhinoplasty implant material containing a cross-linked human acellular dermal matrix has been introduced by Yang et al. (2018), and their clinical experiences have been described. The outpatient clinic assessed clinical outcomes and complications. Two independent plastic surgeons examined preoperative and postoperative photographs to assess contour changes. Six questions concerning aesthetics and function were administered to patients at the outpatient clinic. Both autogenous and alloplastic materials have advantages with cross-linked human ADM. There are no complications associated with the surgical results. As a result, it is an ideal alternative implant material for rhinoplasty augmentation of the dorsal aspect [97]. Li et al. (2014) performed nasal augmentation rhinoplasty using an autologous dermis graft and an alloplastic implant. Translucency and thinning of the skin over alloplastic implants are other side effects of alloplastic implants in addition to skin thinning. Alloplastic implants should be used in conjunction with autologous dermal grafts during rhinoplasty augmentation to minimize the impact of these complications. Chinese patients were satisfied with the results of this procedure. A combination of alloplastic implants and autologous dermis grafts is the most effective method of nasal augmentation, particularly when patients have thin tips [98].

#### Expanded tetrafluoroethylene (Gore-Tex)

Since silicone implants became the most widely used alloplastic implant, Gore-Tex implants have become more popular. A porous implant composed of fluorine and carbon molecules, Gore-Tex has pores that allow connective tissue, such as collagen, capillaries, and fibroblasts, to grow. Infections are increased as a result, increasing the stability of the implant. Gore-Tex’s soft texture makes patients less likely to experience discomfort and see unnatural implant contours through their skin. Despite this, Gore-Tex’s volume decreases after insertion, which is a disadvantage. The removal of Gore-Tex implants is also more complex than silicone implants. According to surgeons, gore-Tex-related complications are most often caused by infections or delayed inflammation. Several debates have taken place between proponents of silicone and Gore-Tex. As a result of their ability to be carved into various shapes and sizes, silicone implants could potentially generate the most aesthetically pleasing results for an augmentation rhinoplasty. The capsules, however, may contract, and migration may be more frequent. However, Gore-Tex implants have a lower likelihood of forming capsules and migrate slower, the operation is challenging, and the chance of infection is slightly higher [73]. The Sili-Tex implant has an ePTFE-wrapped outer surface and an inner silicone core. With the silicone core in the middle and ePTFE on the outside, the implant height is maintained, and tissue ingrowth reduces implant mobility. It has biocompatibility and minimal foreign body reaction, and it is made from silicone and polytetrafluoroethylene. In addition to extrusions, uneven surfaces, infections, recurrent swellings, and seromas, complications with Gore-Tex are also increasing [74].. The double “V” carving technique has been proposed by Bai and coworkers (2020) as a method for improving nasal dynamic mobility. An incision is made on either side at the junction between the mobile and fixed parts of the nasal dorsum. The incision has a 25- to 45° angle, which makes up one-third of the prosthesis's width [99].

#### Silicone

Silicone cannot be reabsorbed or deformed. Silicone is also an alloplastic material. The non-porous nature of silicone makes it easy to sterilize with antiseptic solutions. Porous implants, however, are more likely to cause infection. Pores more significant than 1 mm can allow bacteria to enter, whereas pores more significant than 30 mm are required for macrophages. Silicone rubber also comes in different consistencies. The host reacts only minimally to rubber butyl, which is chemically inert. Silicone rubber is prone to compression and deformation. Since it maintains its shape between 55C and 1400C, it may be autoclaved without damaging its qualities. Silicone implants come in several styles but do not fit every nose. Asia has increasingly relied on silicone implants, especially L-shaped ones. Before and after surgery, each implant is customized for the patient. This silicone implant’s smooth nasal dorsal contour is one of its significant advantages. A silicone L-shaped implant can augment the nasal dorsum and tip at the same time. Asian patients have weak septums, small cartilaginous frameworks, and thick, soft tissue envelopes, making L-shaped silicone an attractive option [100]. A wide range of complications are associated with silicone, depending on the patient population. Extrusions occur at a rate of 2 to 4%, infections at 4%, and displacements at 3%. There are a variety of complications associated with silicone augmentation, including extrusion, displacement, movement, and infection. The silicone implant may become displaced or move if it is inserted too superficially. This can be prevented by placing the silicone below the nasal periosteum. Silicone rhinoplasty can also lead to infection. An ocular vestibule and a nasal cavity must be sterilized thoroughly before surgery to reduce infection. It is essential to protect mucous membranes from damage during surgery. Increasing infection rates through aggressive anatomical modifications, such as osteotomies, is possible. Inadequate implant design can also cause aesthetic problems. A silicone replacement should be performed if there is deviation or displacement. When silicone is removed from the nose, there is no need for additional augmentation in some patients because of capsule formation, which can produce sufficient volume. It is essential to carefully manage the capsule surrounding the silicone DA implant during revision surgery. Capsules can be left in place if they don't cause an aesthetic deformity and do not cause an infection. To avoid damaging the SSTE, the surgeon should carefully trim the capsule just beneath the skin and over the implant before removing it. Using a microdebrider for sinus surgery is an effective way to remove capsules with delicate precision. A capsular contracture around the implant usually causes this phenomenon. It can also occur after implant removal due to progressive scar contracture. Additionally, after long-term use, silicone implantation can cause cartilage and bone resorption in the nasal framework. Septal reconstruction is usually performed to correct short noses, lateral compartment elongation, tip surgery, and revision dorsal augmentation. Furthermore, it provides sufficient cartilage for both dorsal and tip augmentation, unlike septal or conchal cartilage. Due to previous silicone procedures, an elongated nasal skeleton is often covered with an inelastic SSTE. A short nose correction depends mainly on the skin's condition [32]. The ride-on technique (sutured cartilage or perichondrium onto a silicone surface) also reduces implant extrusion rates and maintains the natural mobility of the tip, according to Agrawal et al. (2015) [101].

#### Other grafts

In addition to silicone and ePTFE, several other synthetic implants have less satisfactory results. In a positive light, this tissue reaction may reduce the likelihood of implant migration, as supramid resembles Mersilene and has a high resorption rate. The Mersilene synthetic implant is another durable and contourable synthetic implant made of polyethylene terephthalate. It has been reported that infected materials have a 4% infection rate, with half of these cases being removed due to bacterial colonization. Its disadvantage is that excessive fibroblast ingrowth makes removal difficult. While Mersilene has a lower resorption rate than Supramid, it has often been surpassed by ePTFE due to its lower infection risks and easier removal [2]. Yap et al. (2011) studied patients with DA performed with ePTFE and found that 0.85% had visible implants or an overly elevated bridge. Complications with the ePTFE group included a prominent contour of the implant. Patients with thin skin might have been more susceptible to this sequela due to improperly carved implants. As a result of the soft nature of ePTFE, two patients also experienced displacement. Researchers might have predisposed these patients to this outcome because they undermined the soft-tissue envelope in their rhinoplasty procedure rather than creating a tight pocket for the implant [102].

An evaluation of silicon-polytetrafluoroethylene composite implants for Asian rhinoplasties was performed by Zelken et al. (2017). One hundred forty-nine women and 18 men, aged 19 to 72, were undergoing surgery. A composite implant measured 3.8 to 4.5 cm long and was 1.5 to 5 mm thick. A septum, concha, or both were used to refine tips. A total of 19 glabellar augmentations were performed. After 6.0 months of follow-up, they found no difference in findings. An infection, erythema, and malposition were among the 19 complications. Four patients did not feel satisfied, citing inadequate correction of their dorsal height. A revision rate of 8.8% was observed; 7 of the 12 revisions were associated with malpositions or deviations. Extrusion or step-off of implants was not observed. Infection rates after primary rhinoplasty were higher than after secondary rhinoplasty, but no differences in outcomes were found. The use of I-shaped silicone-PTFE composite implants in Asian patients can be performed both as primary and secondary augmentations of the nose. Based on early outcomes data, compliance rates are comparable to PTFE alone [103].

A 14-year experience with ePTFE procedures has been reported by Wei et al. (2018). They described the technique and discussed the outcomes, patient selection, and complications associated with the procedure. An ePTFE implant was used in all cases to extend the nose. Using 3-dimensional simulation technology, nasal lengths were measured before and after surgery. The outcomes and complications were analyzed, as well as any underlying reasons. Surveys were used to gauge patient satisfaction. When dealing with patients with short noses, ePTFE implantation can accomplish nasal elongation. Patient acceptance is so high that it is attributed to the results’ reliability and the absence of donor site morbidity. Success requires careful selection of patients and meticulous surgical technique [104]. PTFE polymers and carbon fibers combine to make Proplast, another comparable synthetic option. Silicone implants can be shaped similarly to Proplast II, which provides versatility. Despite its increased porosity, the material is susceptible to fragmentation and collapse. Consequently, Proplast II is not recommended for use in cases that require significant structural support [2]. For rhinoplasty with e-PTFE, researchers reported a 3.0% complication rate, while Conrad and Gillman13 reported a 4.8% rate. This is similar to what researchers observed with DA using e-PTFE, which had an overall complication rate of 4%. As previously reported, infection occurred at 3.2 or 3.5% of rhinoplasties. However, the infection rate in their patient series was only 0.6%, perhaps because most rhinoplasties were performed openly with copious irrigation before and after the procedure. Among the e-PTFE group, the senior author had the most noticeable implant contour complication. The sequelae might have been caused by poorly carved implants in thin patients. Another common complication was displacement due to the soft nature of e-PTFE. Also, the wide undermining of the SSTE may have created a loose skin pocket instead of preventing implant displacement. Several studies have reported that ePTFE is associated with a lower complication rate in primary cases than in revision cases. Their experience shows primary and revision cases have similar complication rates [32]. DA rhinoplasty can also use porous polyethylene (Medpor); however, it is reported to have higher complications than other alloplastic options. The extrusion rate is between 3.1% and 10.7%, and the infection rate is between 1% and 6.25% for porous polyethylene. According to some surgeons, porous polyethylene implants may have the advantage of excellent tissue in-growth but can be detrimental if they need to be removed for infection or displacement [31]. There are several types of synthetic implants, but Medpor is a high-density polyethylene implant with interconnected pores. Polyethylene makes up 54% of the final material, and pore space makes up 46%. Its microstructure allows vascularized tissues and collagen to grow expeditiously, creating a robust and stable construction. The Medpor implant has shown favorable use in nasal DA due to its ease of contouring, biocompatibility, and general resistance to infection, resorption, and distortion. Despite its advantages, this porous polyethylene implant has some disadvantages, including its rigidity, which makes it appear unnatural, and its irregular surface, which makes insertion difficult [2]. El-Sabbagh et al. (2017) augmented the dorsal nasal septum using postauricular mastoid fascia. Dorsal nasal augmentation was performed on ten patients during their study. Steri-strips and external nasal splints were used to fix the fascia over the mastoid area. Most of the patients were females, except for one case. In five cases, the operation was done due to ethnic causes, and in five cases, it was done due to posttraumatic deformities. Healing of donor sites was uneventful. The grafts were photographed digitally and followed up for 9 months after surgery. The donor site of mastoid fascia is hidden, which makes it a reliable method. A conchal graft can also be performed here if necessary [105].

### The effects of tissue engineering (stem cells, materials, and growth factors) in rhinoplasty

Tissue engineering alters cartilage shape and functionality by targeting the cartilage’s primary cells, the chondrocytes. Chondrocyte precursor cells are seeded onto 3D scaffolding and transplanted to patients in vitro or in vivo for cartilage tissue engineering. Anatomically, histologically, and mechanically, neocartilage constructs resemble human cartilage. As well as 3D printed metal devices, 3D printed tools, and, in particular, 3D bioprinted bones and cartilages, there have been several applications for 3D bioprinting. Bioprinting techniques have been used to fabricate native organ and tissue-like scaffolds using 3D printing techniques [10, 58, 65, 66, 106]. 3D printing is an emerging technology in tissue engineering and regenerative medicine because it can create custom implants through CAD/CAM processes. Preoperative consultations for rhinoplasty patients use virtual surgery simulation software to determine a reconstructed nasal shape. The differences in shape and size can be calculated digitally, and customized nasal implants can be produced using CAD/CAM technology and 3D printing. Because virtual simulation software uses computer graphics to provide a postoperative facial model, it is possible to predict the revised nose shape and balance. The shape of the nose would be achieved with minimal error through 3D printing because the printed object coincides with the designed object in shape and size. The 3D printing technique may also be used to construct various materials by freeform fabrication, which can be used to regenerate specific tissues using biomaterials. A variety of complications can result from traditional synthetic nasal implants. In addition to being made of safe, biodegradable materials, 3D-printed nasal implants are designed to meet the specific shape of a patient [20, 58, 107, 108]. Hydrophilic, biocompatible, biodegradable, and nonimmunogenic biomaterial scaffolds should be able to contain chondrocytes and stem cells. According to studies, alginate hydrogels possess good chondrogenic properties but cannot be sutured or implanted directly in the nose due to their lack of properties. A study demonstrated that collagen sponges and polyvinyl alcohol can manufacture scaffolds with precise control using electro-spinning and 3D printing [21, 59, 106]. Various hydrogel materials have been used as scaffolds for 3D cell cultures. For example, silk fibroin, alginate, fibrin, and dECM-derived hydrogels mimic ECM and stimulate cell growth. Tissue-specific biochemical cues found in DECM-derived hydrogels exhibit excellent regenerative properties. Despite their capacity to support themselves, dECM-derived hydrogels have low viscosity and mechanical properties, limiting their use in large or complex 3D structures [107, 109–111]. Differentiation and replication of stem cells are potent processes. Compared to embryonic or fetal stem cells, MSCs have less tumorigenic properties than chondrocytes. Several readily accessible tissues, such as umbilical cord blood, adipose tissue, and synovium, contain these cells. In vitro, BM-MSCs can be differentiated into several generations when arranged close to the central sinus. The BM-MSCs in the 3D culture environment undergo chondrogenesis by being stimulated by various growth factors [19, 22, 106]. All are effective when implanted in scaffolds for cartilage production, including mature chondrocytes, stem cells derived from adipose tissue, stem cells derived from bone marrow, and growth factors. An in vitro cartilage experiment must mimic the in vivo condition by controlling the microenvironment. Transforming growth factors (TGFs), bone morphogenic proteins, and fibroblast growth factors are the most common growth factors that support mature tissue formation. Chondrogenesis is promoted by all of them [19, 106].

Wiggenhauser et al. (2019) studied polycaprolactone-based implants for their regenerative potential in vivo as an alternative to autologous cartilage grafting during rhinoplasty. Two groups of minipigs received implants in the nasal dorsum and were allowed to remain in situ for 2 and 6 months, respectively. An immunostaining and histological examination of the implant was performed following harvest. Polycaprolactone strut dimensions were further measured. A 6-month in vivo study of polycaprolactone-based implants found they helped augment the nasal dorsum in a regenerative and stable way. Polycaprolactone implants for nose augmentation could provide an alternative to autologous cartilage grafts [112]. Mendelson et al. (2014) developed a bioactive scaffold that recruited cells and induced chondrogenesis in the nasal dorsum vivo. On a porous poly PLGA base, alginate encapsulates gelatin microspheres containing cytokines. Several doses of recombinant human TGF-3 were loaded into gelatin microspheres, with PBS as a control. As part of the first step, they implanted bilayered scaffolds on top of a shaved surface of native nasal cartilage to induce cell migration. Various histological staining techniques assessed tissue formation and chondrogenesis within PLGA scaffolds. As the first cartilage tissue engineered through cell homing, it could be used for augmentative and reconstructive rhinoplasty since it combined autologous tissues derived from cytostatic factors with biomaterial scaffolds [113].

**Fig. 3:**
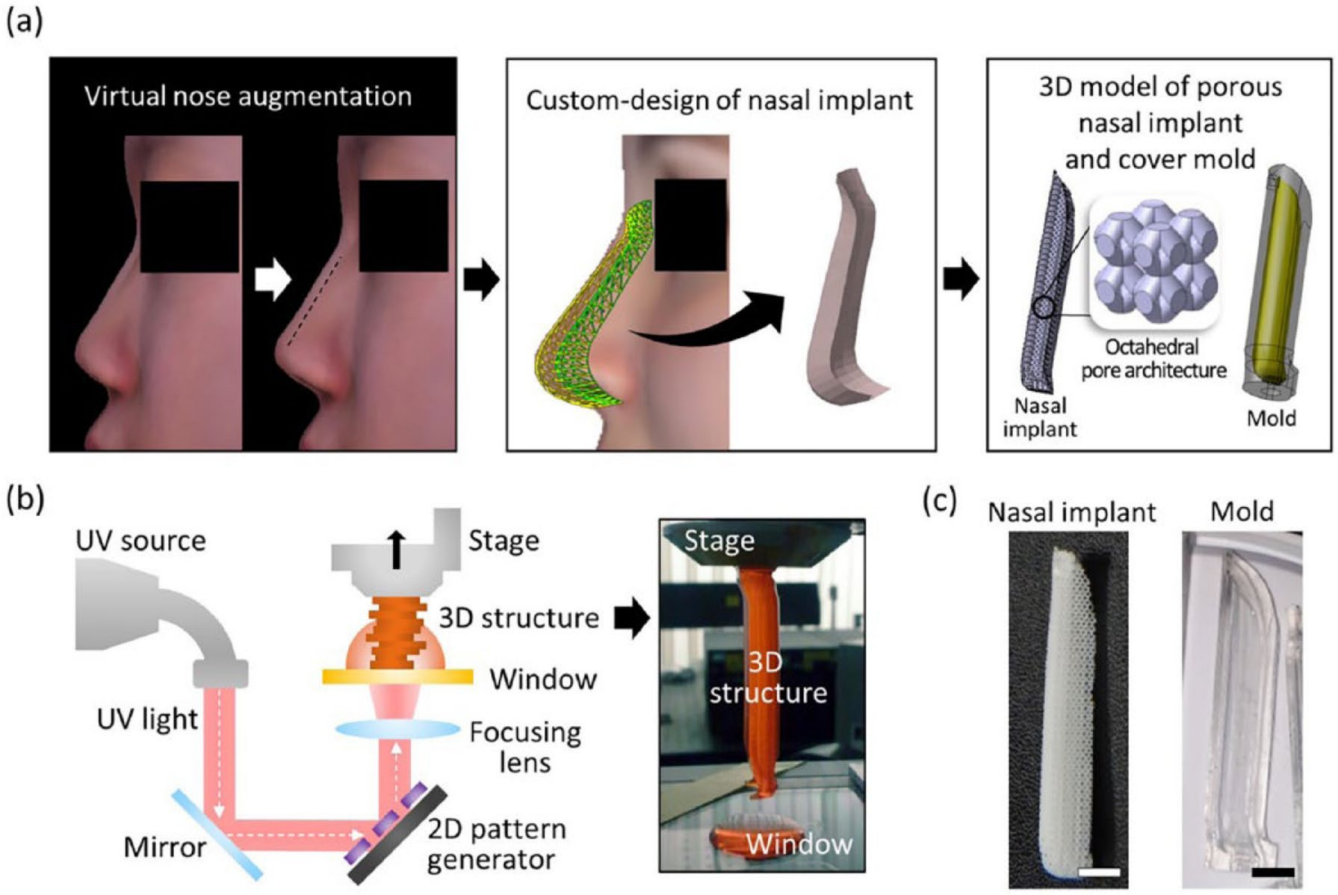
Three-dimensional printing of a patient-customized nasal implant with computer-aided design software. Developing the nasal implantmodel according to the patients custom design. The difference between the preoperative and postoperative noses was calculated usinggeometrical shapes. The geometric difference generated a 3D solid model. A nasal implant model was modified to incorporate an octahedralpattern architecture, and a cover mold model was created based on the nasal implant model (**a**). The pMSTL system is outlined in this schematic (**b**).the patient-specific OrmoComp cover mold and fabricated PCL nasal implant (**c**). Reprinted without further permission from ref [107] from the SAGE and Open Access pages

Yi et al. (2019) used 3D printing and cell-laden hydrogel injection to engineer an augmentative rhinoplasty nasal cartilage implant. Using an algorithm to create the custom nose implant, a 3D model was generated from the virtual preoperative and postoperative nose shapes. CAD software was used to model the interior architecture of an octahedron in the nasal implant model. Figure 3a illustrates a nose that has been augmented. The nasal graft model for this study was generated using the data extracted from the modified and original nasal models. Figure 3b shows a 3D construct built using the pMSTL system. Figure 3c shows an implant fabricated with PCL and an OrmoComp cover mold designed specifically for each patient. The previous process led to the creation of the sacrificial mold model. The cell-laden hydrogel was injected into the nostril implants with the help of a cartilage-derived hydrogel to create a mixture of hASCs and the hydrogel. Human adipose stem cells were differentiated into chondrogenic lineages in cartilage-derived hydrogels in vitro. After implanting engineered nasal cartilage into the mouse subcutaneous region, 3D-printed tissue resembling native tissues formed. Engineered nasal cartilage implants can regenerate cartilage from synthetic and autologous grafts. Their study combines CAD, 3D printing, and cartilage-derived hydrogel, so they anticipated that the process could generate other tissue implants. Using cartilage-derived hydrogel, they engineered nasal cartilage with human adipose-derived stem cells and observed the expression of pronounced chondrogenic markers. For 12 weeks, the engineered subcutaneous nasal cartilage maintained its exquisite shape and structure and striking cartilaginous tissue formation in the subcutaneous region. Aside from producing implants from other types of tissue, they expected that the developed process would help develop other types of implants, including implants made of tissue-derived hydrogels and computer-aided design [107]. According to Peas et al. (2019), their technique combined diced cartilage-fascia grafts with PRP. The procedure used diced cartilage wrapped in a deep-temporal fascia and a chrysalis sleeve for dorsal augmentation rhinoplasty. Using PRP fractions extracted from blood centrifuged, diced cartilage grafts were delivered. A fibrin glue solution was applied over the chrysalis graft using PPP and middle platelet layers instead of stitches. They demonstrated a chrysalis-like technique for dorsal augmentation using diced cartilage, PRP grafts, and deep temporal fascia grafts. An augmentation of the nasal bridge using this technique is non-complicated and minimizes graft resorption [114]. In Castro-Govea et al. (2023), micrografts enriched with ASCs are described as an alternative to conventional nasal modeling. Nanofat was separated from microfat using this technique. They isolated ASCs using nanofat, and enriched microfat with ASCs was used to model nasal structures. Lipoinjectable material was injected into the nasal dorsum in a supraperiosteal plane. An injection of liposomes was carried out through a retrolabial access. ASC-enriched micrografts are an appealing and innovative way to create nonsurgical nasal models. A surgical rhinoplasty will never replace this technique. Patients can return to daily routines after a minor procedure [115].

## Limitations

DA rhinoplasty, a surgical procedure aimed at enhancing the dorsal profile of the nose, has evolved significantly over the years. Despite advancements in surgical techniques, there are inherent limitations that practitioners must acknowledge. This narrative review aims to explore the current techniques in DA rhinoplasty while shedding light on their associated limitations. The limitations of surgical techniques can be categorized as cartilage graft resorption: one of the primary materials used in DA is autologous cartilage grafts. However, a standard limitation is the potential for graft resorption over time. This resorption can compromise the achieved augmentation and lead to a less stable and durable outcome. Graft visibility and palpability: While autologous grafts are often preferred for their biocompatibility, their visibility and palpability under the skin can be limited. This can result in an unnatural appearance and discomfort for the patient. Limited graft availability: The amount of autologous cartilage available for grafting is finite. In cases where extensive augmentation is required, the scarcity of donor cartilage may limit the surgeon's ability to achieve the desired result. Challenges with diced cartilage: Some surgeons opt for diced cartilage wrapped in fascia as a grafting material. However, the unpredictability of how diced cartilage behaves postoperatively and the potential for contour irregularities are significant limitations. Complications with synthetic implants: The use of synthetic implants in DA poses its own set of challenges. The patient-related limitations can be categorized as follows: Ethnic and anatomical variations: DA rhinoplasty may present distinct challenges in patients with diverse ethnic backgrounds due to variations in nasal anatomy. Achieving a harmonious and natural result becomes more complex when considering these differences. Patient expectations: unrealistic patient expectations can be a limitation in DA rhinoplasty. Meeting subjective aesthetic goals can be challenging despite the surgeon’s best efforts, leading to patient dissatisfaction [116]. Postoperative swelling and healing: The resolution of postoperative swelling and the variability in individual healing responses contribute to the challenge of accurately predicting the final aesthetic outcome. Patients must understand that the immediate postoperative appearance may differ from the ultimate result. Psychological impact: like any cosmetic surgery, rhinoplasty can psychologically impact patients. Unrealistic expectations, coupled with postoperative changes, may lead to emotional distress, emphasizing the need for comprehensive preoperative counseling.

The surgical approach limitations can be categorized as follows: Revision surgery challenges: DA rhinoplasty is not exempt from the possibility of revision surgery. However, each subsequent surgery increases the complexity and potential for complications. Scar tissue formation and altered anatomy from previous procedures can limit the success of revisions. Risk of overaugmentation: striking the right balance in DA is crucial. Overaugmentation can lead to an unnatural appearance, impairing nasal function and causing dissatisfaction. Achieving a nuanced and tailored result requires a thorough understanding of facial aesthetics. Nasal function compromise: augmentation procedures may inadvertently compromise nasal function. Altered airflow dynamics, nasal valve collapse, or changes in sensation are potential limitations that must be carefully considered during the surgical planning phase.

The technological limitations can be categorized as follows: imaging accuracy: preoperative imaging plays a vital role in surgical planning. However, the accuracy of imaging technologies in predicting postoperative outcomes is not absolute. Surgeons must navigate the challenge of translating two-dimensional images into three-dimensional surgical reality. Limited predictive modeling: the lack of comprehensive predictive models for DA outcomes remains a limitation. Surgeons rely on their experience and artistic judgment, making the procedure more of an art than an exact science. Advancements in materials and technology: while advancements in surgical materials and technology continue to enhance the field of rhinoplasty, staying abreast of these developments can be challenging. The rapid evolution of techniques may outpace the assimilation of new methods into clinical practice. In conclusion, DA rhinoplasty, while offering transformative results, is not without its limitations. Surgeons must navigate challenges related to graft materials, patient expectations, surgical approaches, and technological constraints. Acknowledging these limitations is essential for fostering realistic expectations, refining surgical techniques, and advancing the field to meet the evolving needs of patients seeking nasal enhancement. Continued research and collaborative efforts within the medical community are paramount to overcoming these limitations and improving DA rhinoplasty’s overall efficacy and safety.

## Future direction

DA rhinoplasty, a surgical procedure aimed at enhancing the height and profile of the nasal dorsum, has evolved significantly over the years. As of the latest review, various techniques have been employed to achieve optimal results regarding aesthetics and functionality. However, the field is dynamic, and advancements continue to reshape the landscape of DA rhinoplasty. In this narrative review, researchers will explore the current DA rhinoplasty techniques and discuss future directions that may further refine and innovate this cosmetic procedure.

### Traditional techniques

Traditional DA rhinoplasty techniques involve using autologous grafts, such as septal cartilage, costal cartilage, or ear cartilage. These grafts are shaped and positioned to augment the nasal dorsum, providing the desired height and contour. While these methods have proven effective, there are limitations, such as donor site morbidity, availability of graft material, and the potential for absorption or warping over time.

### Alloplastic materials

Alloplastic materials, such as silicone implants or high-density porous polyethylene (HDPE), have become an alternative to autologous grafts. These materials offer a readily available and easily moldable solution, reducing operative time and minimizing donor site complications. However, concerns about long-term complications, such as infection, extrusion, or displacement, have led to ongoing research to improve these materials’ biocompatibility and long-term safety.

### Grafting techniques

Recent advancements in grafting techniques have focused on improving the stability and viability of grafts used in dorsal augmentation. Engineered tissues and acellular dermal matrices have emerged as potential alternatives to traditional grafts. These materials aim to scaffold tissue ingrowth, promoting long-term stability and integration. Further research is needed to optimize these techniques and assess their long-term outcomes.

### Three-dimensional imaging and printing

Integrating three-dimensional (3D) imaging and printing technologies can revolutionize DA rhinoplasty. Surgeons can now create patient-specific models and simulate surgical procedures, allowing for meticulous preoperative planning. 3D-printed implants tailored to the patient’s anatomy offer a precise and customizable solution, reducing the risk of asymmetry and enhancing overall surgical outcomes. As technology advances, the affordability and accessibility of 3D printing may become more widespread in clinical practice.

### Biotechnology and regenerative medicine

The field of regenerative medicine holds promise for DA rhinoplasty. Research into tissue engineering and stem cell therapies may lead to the development of bioengineered nasal implants that mimic natural tissues. These implants could potentially overcome the limitations associated with traditional grafts and alloplastic materials. While this area is still in its infancy, ongoing studies may pave the way for novel, biocompatible solutions in the future.

### Minimally invasive techniques

Advancements in minimally invasive techniques have been a prominent trend in various surgical fields, and DA rhinoplasty is no exception. Endoscopic approaches allow smaller incisions, reduced scarring, and quicker recovery times. Using injectable fillers, such as HA or fat grafts, presents a non-surgical alternative for dorsal augmentation. While these minimally invasive options may suit certain patients, their long-term effectiveness and safety require further investigation.

### Augmented and virtual reality in surgical training

The integration of virtual reality and augmented reality (AR) in surgical training has the potential to enhance the skills of surgeons performing DA rhinoplasty. VR simulations can provide a realistic, risk-free environment for practicing and refining surgical techniques. AR systems can overlay digital information onto the surgeon’s field of view during the procedure, offering real-time guidance and enhancing precision. As these technologies become more sophisticated, they may be crucial in training the next generation of rhinoplasty surgeons.

### Patient-centered outcomes and satisfaction

Future directions in DA rhinoplasty should focus on technical advancements and prioritize patient-centered outcomes and satisfaction. Patient-reported outcomes, quality-of-life assessments, and psychological well-being should be integral components of research in this field. Understanding the long-term impact of DA on patients’ lives will contribute to developing more holistic and patient-specific approaches.

### Ethical and cultural considerations

As plastic surgery evolves, ethical considerations surrounding DA rhinoplasty become increasingly important. Surgeons must consider the cultural and societal implications of altering nasal aesthetics. Ethical guidelines and cultural sensitivity should be incorporated into decision-making to ensure patients' desires align with medical principles and societal norms.

## Conclusion

The future of DA rhinoplasty is poised for exciting developments. The landscape of this cosmetic procedure is evolving rapidly from integrating cutting-edge technologies like 3D printing and virtual reality to exploring regenerative medicine and minimally invasive techniques. As research continues to push the boundaries of what is possible, the emphasis should remain on achieving optimal aesthetic outcomes and ensuring patients' safety, satisfaction, and well-being. Surgeons, researchers, and policymakers must collaborate to navigate the ethical considerations and cultural nuances of altering facial aesthetics. The future of DA rhinoplasty holds the promise of more personalized, precise, and patient-centric approaches, ultimately redefining standards in the field of cosmetic surgery.

## Data Availability

Not applicable

## Abbreviations

PRP: Platelet rich plasma
DA: Dorsal augmentation
MISTA: Microfat-infused soft tissue augmentation
FEM: Finite element method
TF: Temporalis fascia
iPRF: Injectable PRF
aPRF: Advanced PRF
GDCG: Glued diced cartilage grafts
DCIF: Diced cartilage in fascia
OCGs: One-block concentric carving cartilage grafts
ROE: Rhinoplasty outcomes evaluation
PRF: Platelet-rich fibrin
PAFG: Posterior auricular fascia grafts
IHCC: Irradiated homologous cartilage
TPFL: Tutoplast-processed fascia lata
PCS: Porcine cartilage-derived substance
CT: Computed tomography
CBCT: Cone-beam computed tomography
DTF: Deep temporal fascia
ACAS: Autogenous control augmentation system
PHDPE: Porous high-density polyethylene
HA: Hyaluronic acid
CaHA: Calcium hydroxyapatite
ECM: Extracellular matrix
dECM: Decellularized extracellular matrix
BM-MSCs: Bone marrow-derived MSCs
hASCs: Human adipose-derived stem cells
ASCs: Adipose-derived mesenchymal stem cells
CAD/CAM: Computer-aided design and manufacturing

## References

[1] Wright JM, Halsey JN, Rottgers SA (2023). Dorsal augmentation: a review of current graft options. Eplasty..

[2] Graw GJ, Calvert JW (2022). Dorsal augmentation. Clin Plast Surg.

[3] Gunter JP, Cochran CS, Marin VP (2008). Dorsal augmentation with autogenous rib cartilage. Semin Plast Surg.

[4] Phillips SJ, Carney MJ, Jazayeri HE, Reategui A, Moores C, Prassinos AJ (2023). Dorsal augmentation with the dorsal extension spreader graft. J Craniofac Surg.

[5] Suh MK (2018). Dorsal augmentation using autogenous tissues. Facial Plast Surg Clin North Am.

[6] Toriumi DM (2017). Dorsal augmentation using autologous costal cartilage or microfat-infused soft tissue augmentation. Facial Plast Surg.

[7] La Padula S, Pensato R, Pizza C, Rega U, D'Andrea F, Roccaro G et al (2023) The use of posterior auricular fascia graft (PAFG) for slight dorsal augmentation and irregular dorsum coverage in primary and revision rhinoplasty: a prospective study. Aesth Plast Surg 1-10 10.1007/s00266-023-03571-0 PMC10980619 37626136

[8] Joo YH, Jang YJ (2016). Comparison of the surgical outcomes of dorsal augmentation using expanded polytetrafluoroethylene or autologous costal cartilage. JAMA Facial Plast Surg.

[9] Aldosari B (2023). A modified technique for autologous dorsal nasal augmentation rhinoplasty. Eur Rev Med Pharmacol Sci.

[10] Tahmasebi E, Mohammadi M, Alam M, Abbasi K, Gharibian Bajestani S, Khanmohammad R (2023). The current regenerative medicine approaches of craniofacial diseases: A narrative review. Front Cell Dev Biol.

[11] Cevizci R, Üstün Bezgin S, Çakir B, Kersin B, Bayazit YA (2017). Dorsal augmentation of saddle nose deformity with toothpick-shaped costal cartilage grafts in the secondary septorhinoplasty. J Craniofac Surg.

[12] Chang C, Kong WK (2014). Clinical effectiveness and safety of collagen sheet for dorsal augmentation in rhinoplasty. J Craniofac Surg.

[13] Ciğer E, İşlek A (2022). Inferior Turbinate Bone Graft for Dorsal Augmentation in Rhinoplasty. Indian J Otolaryngol Head Neck Surg.

[14] Kim MJ, Jeon DN, Choi JW, Chan KS (2020). 3D photogrammetric analysis in hump nose correction based on nasal tip projection without dorsal augmentation in Asian rhinoplasty. J Craniomaxillofac Surg.

[15] Park H, Kim Y, Choi JW (2023). Asian rhinoplasty using a thin rib cartilage graft and ultrafine diced cartilage wrapped in fascia: A comparative study between septal cartilage graft and rib cartilage graft. J Plast Reconstr Aesthet Surg.

[16] Taghva M, Mosaddad SA, Ansarifard E, Sadeghi M. Could various angulated implant depths affect the positional accuracy of digital impressions? An in vitro study. Journal of Prosthodontics.n/a(n/a) 10.1111/jopr.13764 37675589

[17] Patil P, Madhav VNV, Alshadidi AAF, Saini RS, Aldosari LIN, Heboyan A (2023). Comparative evaluation of open tray impression technique: investigating the precision of four splinting materials in multiple implants. BMC Oral Health.

[18] Saini RS, Mosaddad SA, Heboyan A (2023). Application of density functional theory for evaluating the mechanical properties and structural stability of dental implant materials. BMC Oral Health.

[19] Hussain A, Tebyaniyan H, Khayatan D (2022). The role of epigenetic in dental and oral regenerative medicine by different types of dental stem cells: a comprehensive overview. Stem Cells Int.

[20] Yazdanian M, Arefi AH, Alam M, Abbasi K, Tebyaniyan H, Tahmasebi E (2021). Decellularized and biological scaffolds in dental and craniofacial tissue engineering: a comprehensive overview. J Mater Res Technol.

[21] Tafazoli Moghadam E, Yazdanian M, Alam M, Tebyanian H, Tafazoli A, Tahmasebi E (2021). Current natural bioactive materials in bone and tooth regeneration in dentistry: a comprehensive overview. J Mater Res Technol.

[22] Soudi A, Yazdanian M, Ranjbar R, Tebyanian H, Yazdanian A, Tahmasebi E (2021). Role and application of stem cells in dental regeneration: a comprehensive overview. EXCLI J.

[23] Keyhan SO, Ramezanzade S, Yazdi RG, Valipour MA, Fallahi HR, Shakiba M (2022). Prevalence of complications associated with polymer-based alloplastic materials in nasal dorsal augmentation: a systematic review and meta-analysis. Maxillofac Plast Reconstr Surg.

[24] Kim CH, Park SC (2018). Homologous tissue for dorsal augmentation. Facial Plast Surg Clin North Am.

[25] Campiglio G, Rafanelli G, Klinger F, Caviggioli F, Giannasi S, Klinger M (2019). Dorsal augmentation with diced conchal cartilage wrapped in retroauricular fascia. Aesth Plast Surg.

[26] Kim YS, Park DY, Cho YH, Chang JW, Choi JW, Park JK (2015). Cultured chondrocyte and porcine cartilage-derived substance (PCS) construct as a possible dorsal augmentation material in rhinoplasty: a preliminary animal study. J Plast Reconstr Aesthet Surg.

[27] Namgoong S, Kim S, Suh MK (2020). Multilayered costal cartilage graft for nasal dorsal augmentation. Aesth Plast Surg.

[28] Namgoong S, Yang JP, Han SK, Jeong SH, Dhong ES (2019). Clinical analysis of nasal bone fracture in patients who have previously undergone dorsal augmentation using silicone implants: a pilot study. Aesth Plast Surg.

[29] Oh JH, Kim ST, Jung JH, Han JH, Choi JY, Kang IG (2018). Height changes of tutoplast-processed fascia lata over time after dorsal augmentation during rhinoplasty. J Oral Maxillofac Surg.

[30] Lee HJ, Bukhari S, Jang YJ (2021). Dorsal augmentation using crushed autologous costal cartilage in rhinoplasty. Laryngoscope..

[31] Malone M, Pearlman S (2015). Dorsal augmentation in rhinoplasty: a survey and review. Facial Plast Surg.

[32] Na HG, Jang YJ (2017). Dorsal augmentation using alloplastic implants. Facial Plast Surg.

[33] Yoo SH, Kim DH, Jang YJ (2022). Dorsal augmentation using a glued diced cartilage graft fashioned with a newly developed mold in Asian rhinoplasty. Plast Reconstr Surg.

[34] Jang YJ, Yoo SH (2019). Dorsal augmentation in facial profiloplasty. Facial Plast Surg.

[35] Erisgin Z, Hizli O, Yildirim G, Sivrikaya C, Sarisoy AB, Avci Y (2023). Use of hyaluronic acid matrix in dorsal augmentation rhinoplasty. Biotech Histochem.

[36] Zheng R, Wang X, Wang H, You J, Xu Y, Zhang X (2023). Improvement of nasal dorsal onlay graft appearance after augmentation rhinoplasty with costal cartilage for thin-skinned patients. Aesth Plast Surg.

[37] Wang F, Chen L, Jin S, Hu B, Chen W, Wang J (2023). Study on the influence of buried thread nasal augmentation on dorsal soft tissue of nose and revision rhinoplasty. Zhongguo Xiu Fu Chong Jian Wai Ke Za Zhi.

[38] Kook WS, Ryu DH, Baek W, Kook HM, Jang YY, Lew DH (2023). Prevention and resolution of silicone implant-related problems in secondary rhinoplasty using a cross-linked human acellular dermal matrix. Plast Reconstr Surg.

[39] Saito T, Lonic D, Lo CC, Tu JC, Hattori Y, Lo LJ (2023) Septal extension graft in cleft rhinoplasty: Patients with secondary unilateral cleft lip nasal deformity. Plast Reconstr Surg. 10.1097/PRS.0000000000011106. Epub ahead of print. PMID: 37797231 10.1097/PRS.0000000000011106 37797231

[40] Zholtikov V, Golovatinskii V, Ouerghi R, Daniel RK (2021). Rhinoplasty: Aesthetic Augmentation With Improvement of Dorsal Aesthetic Lines. Aesthet Surg J.

[41] Apaydin F, Fernández-Pellón Garcia RF, Sahin FF, Rahavi-Ezabadi S (2022). Cartilage Chips in Rhinoplasty. Facial Plast Surg.

[42] Park SC, Nam JS, Lee KI, Lee YW, Park JJ, Ha JG (2022). Effectiveness of cross-linked human acellular dermal matrix in primary and revision augmentation rhinoplasty. J Plast Reconstr Aesthet Surg.

[43] Li J, Sang C, Fu R, Liu C, Suo L, Yan Y (2022). Long-term complications from diced cartilage in rhinoplasty: a meta-analysis. Facial Plast Surg Aesthet Med.

[44] Kazemi Ashtiani A, Moghimi MR, Hafezi F (2022). Perichondrial attached diced cartilage (PADC), a novel graft material for nasal augmentation: 10 years of experience. Aesthet Surg J.

[45] Alqabbani AA, Assiri H, Mulafikh DS, Hudise J, Aldhabaan S, Nassar R (2022). Indications, techniques, and postoperative outcomes of temporalis fascia grafting in rhinoplasty. J Craniofac Surg.

[46] You J, Wu L, Xu Y, Fan F, Wang H (2021). Comma-shaped columellar strut for nasal tip plasty in East Asian rhinoplasty. Aesth Plast Surg.

[47] Abdel-Aty Y, Prasad N, Hall SR, Howard BE (2021). Diced cartilage in fibrin glue for dorsal reconstruction as part of staged paramedian forehead flap reconstruction. J Craniofac Surg.

[48] Wang H, Wu L, Xu Y, Fan F, You J (2021). Bilateral fan-shaped septal extension Struts in East Asian augmentation rhinoplasty. Aesth Plast Surg.

[49] Vila PM, Jeanpierre LM, Rizzi CJ, Yaeger LH, Chi JJ (2020). Comparison of autologous vs homologous costal cartilage grafts in dorsal augmentation rhinoplasty: a systematic review and meta-analysis. JAMA Otolaryngol Head Neck Surg.

[50] Heidari MB, Rasti M, Nadri S, Roozbehani A, Farhang Fallah A, Mahmoudvand H (2020). Comparison between wrapping dice cartilage with temporal fascia and wrapping in alloderm for dorsal nasal augmentation. World J Plast Surg.

[51] Mizuno T (2019). A new technique for augmentation rhinoplasty using hybrid autologous grafts with septal extension grafts in Asian patients. Facial Plast Surg.

[52] Lee W, Kim JS, Oh W, Koh IS, Yang EJ (2019). Nasal dorsum augmentation using soft tissue filler injection. J Cosmet Dermatol.

[53] Kim J, Jung HJ, Shim WS (2018). Corrective septorhinoplasty in acute nasal bone fractures. Clin Exp Otorhinolaryngol.

[54] Jeong JY, Ha Y, Kim S, Yang HJ, Oh SH (2018). Availability and safety of osteotomy in esthetic rhinoplasty of East Asian patients. Ann Plast Surg.

[55] Swaroop GS, Reddy JS, Mangal MC, Gupta A, Nanda BS, Jhunjhunwala N (2018). Autogenous control augmentation system - a refinement in diced cartilage glue graft for augmentation of dorsum of nose. Indian J Plast Surg.

[56] Mehta JS, Zade MP, Nakade DV, Gupta S, Akhila CV (2021). Augmentation rhinoplasty using olecranon bone graft. Natl J Maxillofac Surg.

[57] Clark RP, Pham PM, Ciminello FS, Hagge RJ, Drobny S, Wong GB (2019). Nasal dorsal augmentation with freeze-dried allograft bone: 10-year comprehensive review. Plast Reconstr Surg.

[58] Yazdanian M, Alam M, Abbasi K, Rahbar M, Farjood A, Tahmasebi E (2022). Synthetic materials in craniofacial regenerative medicine: a comprehensive overview. Front Bioeng Biotechnol.

[59] Hakim LK, Yazdanian M, Alam M, Abbasi K, Tebyaniyan H, Tahmasebi E (2021). Biocompatible and biomaterials application in drug delivery system in oral cavity. Evid-Based Complement Alternat Med: eCAM.

[60] Hoang TA, Lee KC, Dung V, Chuang SK (2022). Augmentation rhinoplasty in cleft lip nasal deformity using alloplastic material and autologous cartilage. J Craniofac Surg.

[61] Atespare A, Kara H, Ilter E, Boyaci Z, Çelik Ö, Midi A (2016). Utility of cartilage grafts wrapped with amniotic membrane in dorsal nasal augmentation. J Craniofac Surg.

[62] Cerkes N, Basaran K (2016). Diced cartilage grafts wrapped in rectus abdominis fascia for nasal dorsum augmentation. Plast Reconstr Surg.

[63] El Abany A, Kandathil CK, Castillo N, Abdelhamid AS, Kimura K, Most SP (2023) Outcomes of diced cartilage dorsal augmentation in dorsal aesthetic deformities. Facial Plast Surg Aesthet Med. 10.1089/fpsam.2023.0059. Epub ahead of print. PMID: 37707994 10.1089/fpsam.2023.0059 37707994

[64] Zinser MJ, Siessegger M, Thamm O, Theodorou P, Maegele M, Ritter L (2013). Comparison of different autografts for aural cartilage in aesthetic rhinoplasty: is the tragal cartilage graft a viable alternative?. Br J Oral Maxillofac Surg.

[65] Mosaddad SA, Hussain A, Tebyaniyan H (2023) Exploring the use of animal models in craniofacial regenerative medicine: a narrative review. Tissue Eng Part B, Rev 30 10.1089/ten.TEB.2023.0038 37432898

[66] Khayatan D, Hussain A, Tebyaniyan H (2023) Exploring animal models in oral cancer research and clinical intervention: A critical review. Vet Med Sci 9 10.1002/vms3.1161 PMC10357283 37196179

[67] Yoo SH, Jang YJ (2019). Rib cartilage in Asian rhinoplasty: new trends. Curr Opin Otolaryngol Head Neck Surg.

[68] Ujam AB, Vig N, Nasser N (2023). The 10th Costal Cartilage Graft in Secondary Cleft Rhinoplasty-A Versatile Rib. Facial Plast Surg.

[69] Leach L, Shamil E, Malata CM (2018). Indications and Long-term Outcomes of Open Augmentation Rhinoplasty with Autogenous L-shaped Costal Cartilage Strut Grafts - A Single Plastic Surgeon's Experience. Otolaryngol Pol.

[70] Lee YH, Choi YS, Bae CH, Song SY, Kim YD, Na HG (2022). Crushed septal cartilage-covered diced cartilage glue (CCDG) graft: a hybrid technique of crushed septal cartilage. Aesth Plast Surg.

[71] Lee JS, Lee DC, Ha DH, Kim SW, Cho DW (2016). Redefining the Septal L-Strut to Prevent Collapse. PLoS One.

[72] Ahn TH, Zheng T, Kang HJ, Yoo BJ, Chung JH, Jeong JH (2019). New technique in nasal tip plasty: sandwich technique using cartilage and septal bone complex. Ear Nose Throat J.

[73] Li D, An Y, Yang X (2016). An overview of asian rhinoplasty. Ann Plast Surg.

[74] Na HG, Jang YJ (2020). Use of nasal implants and dorsal modification when Treating the East Asian Nose. Otolaryngol Clin N Am.

[75] Varedi P, Bohluli B (2015). Dorsal nasal augmentation: is the composite graft consisting of conchal cartilage and retroauricular fascia an effective option?. J Oral Maxillofac Surg.

[76] Erol OO (2000). The Turkish delight: a pliable graft for rhinoplasty. Plast Reconstr Surg.

[77] Codazzi D, Ortelli L, Robotti E (2014). Diced cartilage combined with warm blood glue for nasal dorsum enhancement. Aesth Plast Surg.

[78] Daniel RK (2008). Diced cartilage grafts in rhinoplasty surgery: current techniques and applications. Plast Reconstr Surg.

[79] Tasman AJ, Suárez GA (2015). The Diced Cartilage Glue Graft for Radix Augmentation in Rhinoplasty. JAMA Facial Plast Surg.

[80] Varedi P, Bohluli B (2015). Dorsal Nasal Augmentation: Is the Composite Graft Consisting of Conchal Cartilage and Retroauricular Fascia an Effective Option?. J Oral Maxillofac Surg.

[81] Kim JH, Jang YJ (2015). Use of diced conchal cartilage with perichondrial attachment in rhinoplasty. Plast Reconstr Surg.

[82] Hudise JY, Aldhabaan SA, Alqabbani AA, Nassar RS, Alarfaj AM (2021). Tubed temporalis fascia for nasal dorsal contouring: a novel technique. ORL J Otorhinolaryngol Relat Spec.

[83] Dong W, Han R, Fan F (2022). Diced cartilage techniques in rhinoplasty. Aesth Plast Surg.

[84] Namanloo RA, Ommani M, Abbasi K, Alam M, Badkoobeh A, Rahbar M (2022). Biomaterials in guided bone and tissue regenerations: an update. Adv Mater Sci Eng.

[85] Naeimi Darestani M, Asl Roosta H, Mosaddad SA, Yaghoubee S (2023). The effect of leukocyte- and platelet-rich fibrin on the bone loss and primary stability of implants placed in posterior maxilla: a randomized clinical trial. Int J Implant Dent.

[86] Beaudoin PL, Carles G (2023). Template for diced cartilage with platelet-rich fibrin (PRF) in rhinoplasty: an easy solution for millimetric camouflage of the full dorsal esthetic unit. Facial Plast Surg.

[87] Fung CY, Kim JH, Liao PH, Jang YJ (2023). Revision rhinoplasty using glued diced costal cartilage shaped with mold for management of complicated silicone rhinoplasty. Aesthet Surg J.

[88] Park P, Jin HR (2016). Diced cartilage in fascia for major nasal dorsal augmentation in Asians: a review of 15 consecutive cases. Aesth Plast Surg.

[89] Kao WP, Lin YN, Lin TY, Huang YH, Chou CK, Takahashi H (2016). Microautologous fat transplantation for primary augmentation rhinoplasty: long-term monitoring of 198 Asian patients. Aesthet Surg J.

[90] Jeong HY, Cho KS, Bae YC, Seo HJ (2022). Simple method of saddle nose correction: a double-layer dermofat graft: case report. Medicine (Baltimore).

[91] Rosengaus F, Nikolis A (2020). Cannula versus needle in medical rhinoplasty: the nose knows. J Cosmet Dermatol.

[92] Rosenberger ES, Toriumi DM (2016). Controversies in revision rhinoplasty. Facial Plast Surg Clin North Am.

[93] Rauso R, Tartaro G, Chirico F, Zerbinati N, Albani G, Rugge L (2020). Rhinofilling with hyaluronic acid thought as a cartilage graft. J Craniofac Surg.

[94] Kustermans L, Mommaerts MY (2017). The hydroxyapatite Turkish Delight: a technical note. Oral Maxillofac Surg.

[95] Mosaddad SA, Rasoolzade B, Namanloo RA, Azarpira N, Dortaj H (2022). Stem cells and common biomaterials in dentistry: a review study. J Mater Sci Mater Med.

[96] Mohammadyari F, Parvin S, Khorvash M, Amini A, Behzadi A, HajEbrahimi R et al (2023) Acellular dermal matrix in reconstructive surgery: applications, benefits, and cost. Front Transplant. 2 10.3389/frtra.2023.1133806 PMC11235262 38993878

[97] Yang CE, Kim SJ, Kim JH, Lee JH, Roh TS, Lee WJ (2018). Usefulness of cross-linked human acellular dermal matrix as an implant for dorsal augmentation in rhinoplasty. Aesth Plast Surg.

[98] Li SH, Liu HW, Cheng B, Xiao LL, Xie GH, Xie B (2014). Combined alloplastic implant and autologous dermis graft for nasal augmentation rhinoplasty in Asians. Aesth Plast Surg.

[99] Bai SS, Li D, Xu L, Duan HC, Yuan J, Wei M (2020). A novel method to enhance dynamic rhinoplasty outcomes: double "V" carving for alloplastic grafts. Ear Nose Throat J.

[100] Kim IS (2018). Augmentation rhinoplasty using silicone implants. Facial Plast Surg Clin North Am.

[101] Agrawal KS, Bachhav MV, Naik CS, Gupta S, Sarda AV, Desai V (2015). "Ride-on" technique and other simple and logical solutions to counter most common complications of silicone implants in augmentation rhinoplasty. Indian J Plast Surg.

[102] Yap EC, Abubakar SS, Olveda MB (2011). Expanded polytetrafluoroethylene as dorsal augmentation material in rhinoplasty on Southeast Asian noses: three-year experience. Arch Facial Plast Surg.

[103] Zelken JA, Hong JP, Chang CS, Hsiao YC (2017). Silicone-polytetrafluoroethylene composite implants for Asian rhinoplasty. Ann Plast Surg.

[104] Wei J, Herrler T, Deng N, Xu H, Shi B, Dai C (2018). The use of expanded polytetrafluoroethylene in short nose elongation: fourteen years of clinical experience. Ann Plast Surg.

[105] El-Sabbagh AH (2017). The use of mastoid fascia for dorsal nasal augmentation. Clujul Med.

[106] Farahani PK (2023). Application of tissue engineering and biomaterials in nose surgery.

[107] Yi H-G, Choi Y-J, Jung JW, Jang J, Song T-H, Chae S (2019). Three-dimensional printing of a patient-specific engineered nasal cartilage for augmentative rhinoplasty. J Tissue Eng.

[108] Yazdanian M, Rahmani A, Tahmasebi E, Tebyanian H, Yazdanian A, Mosaddad SA (2021). Current and advanced nanomaterials in dentistry as regeneration agents: an update. Mini Rev Med Chem.

[109] Mosaddad SA, Yazdanian M, Tebyanian H, Tahmasebi E, Yazdanian A, Seifalian A (2020). Fabrication and properties of developed collagen/strontium-doped Bioglass scaffolds for bone tissue engineering. J Mater Res Technol.

[110] Moghadam ET, Yazdanian M, Tahmasebi E, Tebyanian H, Ranjbar R, Yazdanian A (2020). Current herbal medicine as an alternative treatment in dentistry: in vitro, in vivo and clinical studies. Eur J Pharmacol.

[111] Soufdoost RS, Yazdanian M, Tahmasebi E, Yazdanian A, Tebyanian H, Karami A (2019). In vitro and in vivo evaluation of novel Tadalafil/β-TCP/Collagen scaffold for bone regeneration: A rabbit critical-size calvarial defect study. Biocybern Biomed Eng.

[112] Wiggenhauser PS, Balmayor ER, Rotter N, Schantz JT (2019). In vivo evaluation of a regenerative approach to nasal dorsum augmentation with a polycaprolactone-based implant. Eur J Med Res.

[113] Mendelson A, Ahn JM, Paluch K, Embree MC, Mao JJ (2014). Engineered nasal cartilage by cell homing: a model for augmentative and reconstructive rhinoplasty. Plast Reconstr Surg.

[114] Guisantes E (2019) The chrysalis graft: combination of diced-cartilage-fascia grafts and PRP in augmentation rhinoplasty. Int J Transplant Plastic Surg 3

[115] Castro-Govea Y, García-Garza JA, Vázquez-Lara SE, González-Cantú CM, Chacón-Moreno H, Cervantes-Kardasch VH (2023). Lipoinjection with adipose stem cells for nasal modeling: rhino cell, a highly versatile alternative. Arch Plast Surg.

[116] Niakan S, Mahgoli H, Afshari A, Mosaddad SA, Afshari A (2023) Conventional maxillary denture versus maxillary implant-supported overdenture opposing mandibular implant-supported overdenture: Patient's satisfaction. Clin Exp Dent Res 10 10.1002/cre2.813 PMC10860552 38044541

